# Chronic IL-1β-induced inflammation regulates epithelial-to-mesenchymal transition memory phenotypes via epigenetic modifications in non-small cell lung cancer

**DOI:** 10.1038/s41598-019-57285-y

**Published:** 2020-01-15

**Authors:** Rui Li, Stephanie L. Ong, Linh M. Tran, Zhe Jing, Bin Liu, Stacy J. Park, Zi Ling Huang, Tonya C. Walser, Eileen L. Heinrich, Gina Lee, Ramin Salehi-Rad, William P. Crosson, Paul C. Pagano, Manash K. Paul, Shili Xu, Harvey Herschman, Kostyantyn Krysan, Steven Dubinett

**Affiliations:** 1 Pulmonary and Critical Care Medicine, David Geffen School of Medicine at UCLA, Los Angeles, 90025 California USA; 2 Department of Molecular and Medical Pharmacology, David Geffen School of Medicine at UCLA, Los Angeles, 90025 California USA; 3 Department of Medicine, David Geffen School of Medicine at UCLA, Los Angeles, 90025 California USA; 4 Department of Pathology and Laboratory Medicine, David Geffen School of Medicine at UCLA, Los Angeles, 90025 California USA; 5 VA Greater Los Angeles Health Care System, Los Angeles, California 90025 USA

**Keywords:** Cancer microenvironment, Non-small-cell lung cancer

## Abstract

Chronic inflammation facilitates tumor progression. We discovered that a subset of non-small cell lung cancer cells underwent a gradually progressing epithelial-to-mesenchymal (EMT) phenotype following a 21-day exposure to IL-1β, an abundant proinflammatory cytokine in the at-risk for lung cancer pulmonary and the lung tumor microenvironments. Pathway analysis of the gene expression profile and *in vitro* functional studies revealed that the EMT and EMT-associated phenotypes, including enhanced cell invasion, PD-L1 upregulation, and chemoresistance, were sustained in the absence of continuous IL-1β exposure. We referred to this phenomenon as EMT memory. Utilizing a doxycycline-controlled SLUG expression system, we found that high expression of the transcription factor SLUG was indispensable for the establishment of EMT memory. High SLUG expression in tumors of lung cancer patients was associated with poor survival. Chemical or genetic inhibition of SLUG upregulation prevented EMT following the acute IL-1β exposure but did not reverse EMT memory. Chromatin immunoprecipitation and methylation-specific PCR further revealed a SLUG-mediated temporal regulation of epigenetic modifications, including accumulation of H3K27, H3K9, and DNA methylation, in the *CDH1* (E-cadherin) promoter following the chronic IL-1β exposure. Chemical inhibition of DNA methylation not only restored E-cadherin expression in EMT memory, but also primed cells for chemotherapy-induced apoptosis.

## Introduction

Dysregulated inflammation is recognized as one of the hallmarks of cancer and is involved in tumor initiation, progression, and metastasis^1–3^. Chronic inflammatory conditions, such as chronic obstructive pulmonary disease or ulcerative colitis, are strongly associated with elevated cancer incidence^4–6^. Chronic use of aspirin or other non-steroidal anti-inflammatory drugs reduces mortality of esophageal, colorectal, and lung cancers^7,8^.

Interleukin-1 beta (IL-1β), a proinflammatory cytokine, correlates with tumor progression in non-small cell lung cancer (NSCLC) patients in multiple studies. Wu *et al*. found an elevated level of serum IL-1β in NSCLC patients compared to healthy donors. Elevated IL-1β in these patients is associated with poor survival^9^. A similar conclusion was drawn in an independent study showing that elevated serum IL-1β correlates with poor progression-free survival in NSCLC patients^10^. In another study investigating tumor-associated inflammatory responses in early stage lung cancer, high levels of IL-1β were found to be associated with 3-year mortality in adenocarcinoma^11^. Consistent with these findings, the abundance of IL-1 receptor antagonist is associated with a decreased lung cancer risk in a nested case-control study^12^. Particularly noteworthy, the Canakinumab Anti-inflammatory Thrombosis Outcomes Study (CANTOS) revealed that IL-1β inhibition with the neutralizing antibody canakinumab decreases both the incidence and mortality of lung cancer in patients with atherosclerosis co-morbidities^13^. Although our previous studies and those from others demonstrated that acute IL-1β exposure induces the expression of genes associated with transformation, invasion, and metastasis in multiple malignancies, including lung cancer, the impact of chronic IL-1β exposure, which may be more physiologically relevant, has not yet been defined^14–20^.

Metastasis is the primary cause of cancer-related mortality^3,21,22^. Epithelial-to-mesenchymal transition (EMT) plays a critical role in cancer metastasis because it endows metastatic cells with migratory and invasive properties, apoptosis resistance, and immune evasion^23^. In addition, successful colonization in distant organs requires mesenchymal-to-epithelial transition (MET)^24^. The EMT program can be activated in response to pathologic signals such as inflammatory cytokines in the primary tumor microenvironment (TME). However, the mechanisms by which metastatic cells maintain the mesenchymal properties following detachment from the primary tumor and subsequently regain the epithelial status to form macrometastases are unknown.

Here, we define “EMT memory” as a prolonged EMT program induced by chronic IL-1β exposure in NSCLC cells in which EMT features persist in the absence of the inflammatory signal. We identify a unique pattern of epigenetic modifications that leads to the formation of EMT memory. This study reveals distinct molecular events modulating acute and chronic inflammation, thus enhancing our understanding of the temporal-spatial regulation of EMT during metastasis.

## Results

### Chronic cytokine exposure leads to EMT memory in NSCLC

**Figure 1:**
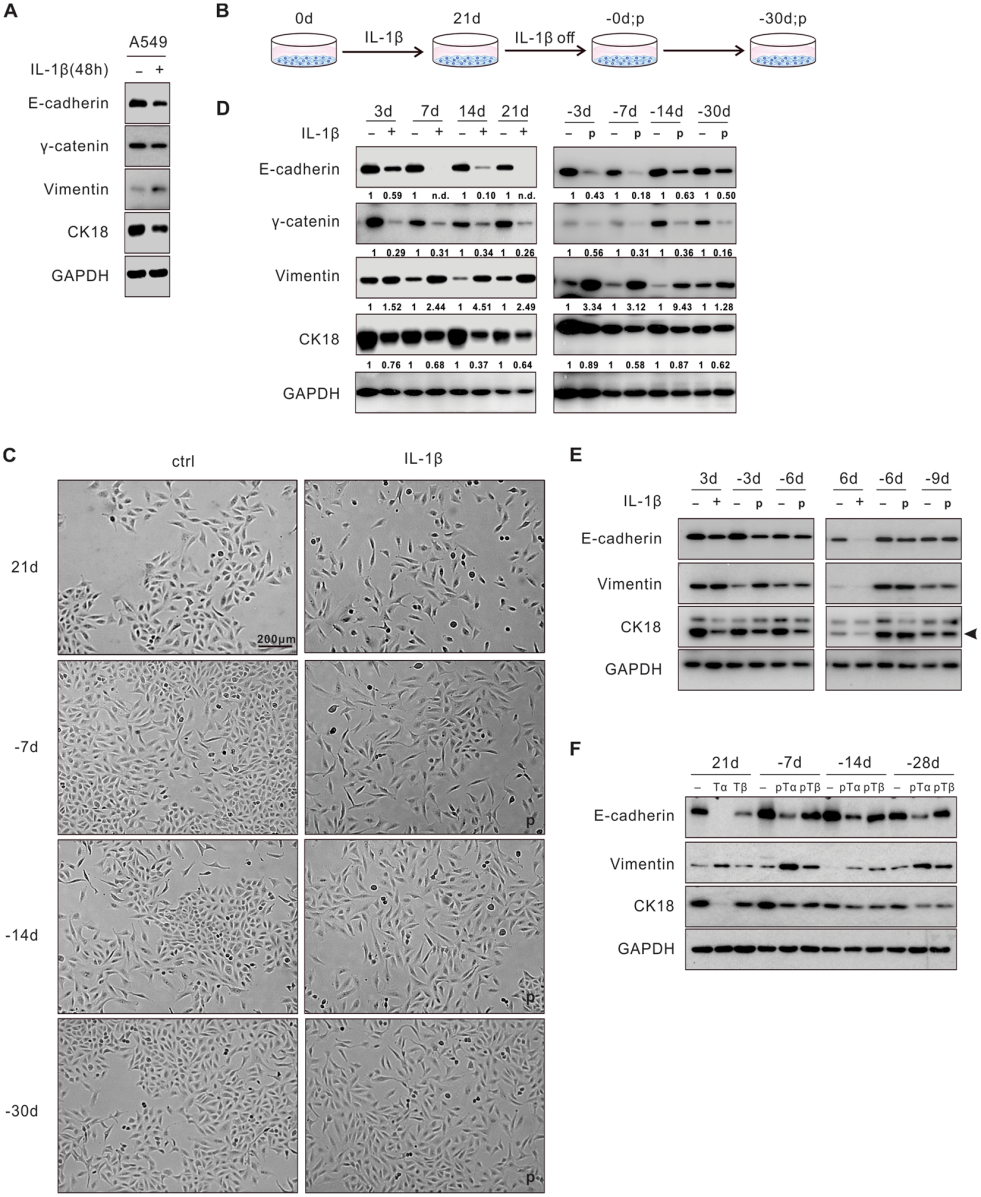
Chronic cytokine exposure leads to EMT memory in NSCLC. (**A**) A549 cells underwent EMT upon 48-hour IL-1β (1 ng/ml) exposure. (**B**) Experimental design for the chronic IL-1β treatment: A549 cells were exposed to IL-1β for 21 days and subsequently cultured for 30 days after IL-1β withdrawal. Cells were split every three to four days with fresh IL-1β in the medium. (**C**) Morphological changes of the untreated and treated A549 cells with IL-1β by bright-field microscopy at different time points. (**D**) EMT markers in A549 cells were determined by immunoblotting following the time course. Quantification of each marker was normalized to the untreated samples. (**E**) EMT was reversed within 6 days upon IL-1β withdrawal following 3 days or 6 days of IL-1β treatment in A549 cells. (**F**) A549 cells chronically treated with TNF-α (10 ng/ml) or TGF-β (5 ng/ml) for 21 days were examined for EMT and EMT memory. “-d”, days after IL-1β withdrawal; “p”, previously treated with IL-1β. “n.d.”, non-detectable. “Tα”, TNF-α; “Tβ”, TGF-β; “pTα”, previously treated with TNF-α; “pTβ”, previously treated with TGF-β. See also Fig. S1.

Because IL-1β and IL-1β-induced genes have been implicated in promoting lung tumor progression in multiple experimental settings and human specimens^10,14–16,25^, we sought to examine whether IL-1β induced EMT in NSCLC. When exposed to IL-1β (1 ng/ml) for 48 hours, A549 lung cancer cells displayed a disaggregated growth pattern with increased cell protrusions, indicating a mesenchymal phenotype (Fig. S1A). Downregulation of several epithelial markers including E-cadherin, gamma-catenin, and cytokeratin 18 (CK18), and upregulation of vimentin, a mesenchymal marker, were confirmed in these cells by immunoblotting (Fig. 1A). Other mesenchymal markers, such as N-cadherin, smooth muscle actin (SMA), and Fibronectin, were also upregulated (Fig. S1B). Because chronic inflammation may play a greater role during tumor development than acute inflammation, we next investigated the impact of chronic IL-1β exposure on these tumor cells (Fig. 1B). When exposed to IL-1β for 21 days, A549 cells acquired a gradually progressing mesenchymal phenotype, as evidenced by changes in both cell morphology and molecular markers (Fig. 1C,D). Of note, following IL-1β withdrawal, these mesenchymal features were maintained for at least 30 days before reverting to the pretreatment epithelial state. We designated this prolonged yet reversible EMT program in the absence of the original inflammatory stimulus “EMT memory.” EMT memory appeared to be IL-1β-exposure time-dependent because shorter exposure time (3-, 6-, or 9-day exposure) did not lead to EMT memory (Figs. 1E and S1C). In addition, a ten-fold increase in IL-1β concentration (10 ng/ml) did not accelerate the EMT memory formation (Fig. S1C**)**.

To determine whether EMT memory was unique to IL-1β stimulation, we chronically exposed A549 cells to TNF-α or TGF-β, two other inflammatory cytokines that are commonly found in the lung TME. Similarly, both cytokines were found to induce EMT memory (Fig. 1F). We also screened an additional ten NSCLC cell lines for IL-1β response and found that two of these cell lines, A427 and H460, had EMT changes in at least two of the examined markers following a 48-hour IL-1β exposure and demonstrated a prolonged EMT phenotype following a 21-day IL-1β exposure (Fig. S1D–F). The EMT memory phenotype in A427 and H460 was evident and endured for 12 to 18 days. This shorter duration of EMT memory may be related to the lack of potential for further mesenchymal transition in these two lines as their baseline E-cadherin expression was not detectable (Fig. S1D,E).

### EMT-associated phenotypes induced by chronic IL-1β exposure are also memorized

**Figure 2:**
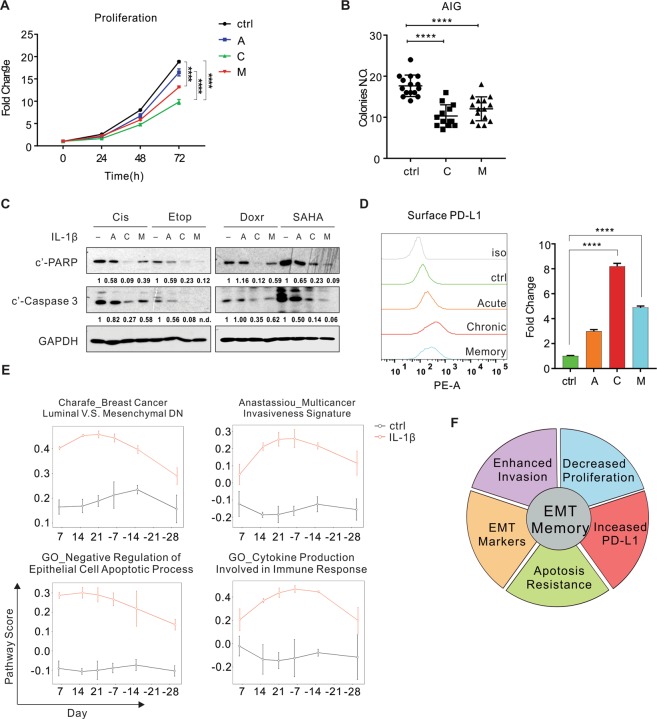
EMT-associated phenotypes induced by chronic IL-1β exposure are also memorized. (**A**) Growth curve of A549 cells with various IL-1β treatments, normalized to the value on day 0 on each condition. (**B**) A dot plot showing numbers of colonies at day 14 after A549 cells were plated for anchorage-independent growth (AIG). Each dot represents a duplicate well. **(C**) Apoptotic markers were evaluated 24 hours after A549 cells were treated with chemotherapy agents. Quantification of cleaved PARP (c’-PARP) and Caspase 3 (c’-Caspase 3) was normalized to samples without IL-1β exposure. (**D**) Surface PD-L1 expression was assessed by flow cytometry. Quantification from 3 replicates is represented in the bar graph. (**E**) Pathway analysis of EMT-associated biological processes at different time points. (**F**) A pie chart summarizing EMT memory phenotypes. “A”, acute IL-1β exposure (72 hours); “C”, chronic IL-1β exposure (≥21 days); “M”, EMT memory (≥7 days after IL-1β withdrawal from chronic exposure). Cis, cisplatin; Etop, etoposide; Doxr, doxorubicin. “n.d.”, non-detectable. All results are reported as mean ± SEM. ****P < 0.0001. See also Fig. S2, Tables S1 and S2.

Given the known pleiotropic effects of EMT, we characterized several key EMT-associated phenotypes in the model of IL-1β exposure in A549 cells. First, the cellular proliferation rate decreased following IL-1β exposure in 2-dimensional cell culture (Fig. 2A). To better assess the tumorigenic potential of these cells, we evaluated the anchorage-independent growth (AIG) in a 3-dimensional assay as previously described^26^. Cells formed fewer and smaller colonies in semisolid medium following both the chronic IL-1β exposure and EMT memory, indicating an impaired potential for solid tumor formation (Fig. 2B). This result is consistent with previous findings showing that mesenchymal cells proliferate more slowly compared to their epithelial counterparts, and MET is required for establishing metastatic lesions^27,28^. Second, IL-1β-treated cells acquired apoptosis resistance to chemotherapy agents, including cisplatin, etoposide, doxorubicin, and vorinostat (SAHA) (Fig. 2C). Third, we evaluated PD-L1 expression because previous studies demonstrated a correlation between a mesenchymal signature and elevated expression of immune checkpoint proteins in human NSCLC^29,30^. We found increased PD-L1 levels in these IL-1β-treated cells (Fig. 2D). Notably, the chronic IL-1β exposure led to more pronounced EMT-associated phenotypes compared to the acute IL-1β exposure. Following the chronic IL-1β exposure, these phenotypes were sustained for at least 7 days after IL-1β withdrawal. In addition, we found chemoresistance and PD-L1 upregulation in H460 and chemoresistance in A427 following the acute IL-1β exposure (Fig. S2A,B).

Given the robust EMT and EMT-associated memory phenotypes in A549 cells, we characterized the underlying molecular events in A549 cells by RNA sequencing. Compared to the untreated control cells, 741 genes were differentially expressed at more than one time point in the presence of IL-1β and formed four clusters (Fig. S2C and Table S1). The vast majority of genes in cluster 1 (upregulated genes) and 4 (downregulated genes) were sensitive to IL-1β, so that their expression returned to the base line immediately upon IL-1β withdrawal. Many of the genes in these two clusters are cytokines, chemokines, and regulatory proteins involved in inflammation and immune response (e.g. *CXCL8*, *CCL2, TGFB1* and *MAP4K1*). However, gene expression in cluster 2 (upregulated genes) and 3 (downregulated genes) exhibited a memory pattern in which the deregulation remained for at least 30 days following IL-1β removal. These genes encode many proteins involved in integrin cell surface interactions, extracellular matrix organization, invasion, and immune checkpoints (e.g. *ITGB3*, *RUNX2, SMAD6*, *FOXA1* and *CD274)*, in addition to the key EMT markers such as *CLDN2* and *CDH1* (Fig. S2C and Table S1).

We then performed the pathway analysis of the whole transcriptome to determine if genes involved in the same pathway revealed concurrent alterations in expression. Among 10,611 gene sets/pathways available in the Molecular Signature Database^31^, 555 were found to be deregulated by IL-1β stimulation. Approximately 48% of these exhibited memory effect and persisted for more than half of their peak activities for 15 days following IL-1β withdrawal, while the remainder of the pathways were identified as IL-1β sensitive pathways as their activities readily reversed following IL-1β withdrawal (Table S2). IL-1β exposure immediately deregulated many signaling pathways, including the AP-1, NF-κB, JNK, and AKT pathways, as well as cellular programs, such as cell migration and differentiation. The activities of these pathways were sensitive to IL-1β (Fig. S2D and Table S2). Consistent with the observed mesenchymal morphologies, genes involved in EMT showed significantly upregulated expression within seven days of treatment, further increased during the chronic treatment, and were maintained at high levels for 30 days following IL-1β withdrawal (Fig. 2E). In addition to EMT, the chronic IL-1β exposure also increased and maintained expression of genes related to cell adhesion, cancer invasion, apoptosis resistance, and production of cytokines involved in immune responses (Fig. 2E and Table S2). In summary, IL-1β induces EMT-associated phenotypes in NSCLC cell lines, including decreased proliferation, enhanced invasion, resistance to cell death, and PD-L1 upregulation, all of which are memorized following chronic IL-1β exposure (Fig. 2F).

Based on the status of IL-1β exposure, we divided EMT into three phases: (i) acute EMT, in which cells are exposed to IL-1β for fewer than 7 days; (ii) chronic EMT, in which cells are exposed to IL-1β for at least 21 days; and (iii) EMT memory, in which cells are cultured in the absence of IL-1β after chronic EMT is established.

### Accumulation of SLUG is indispensable for the establishment of the memory phenotypes

**Figure 3:**
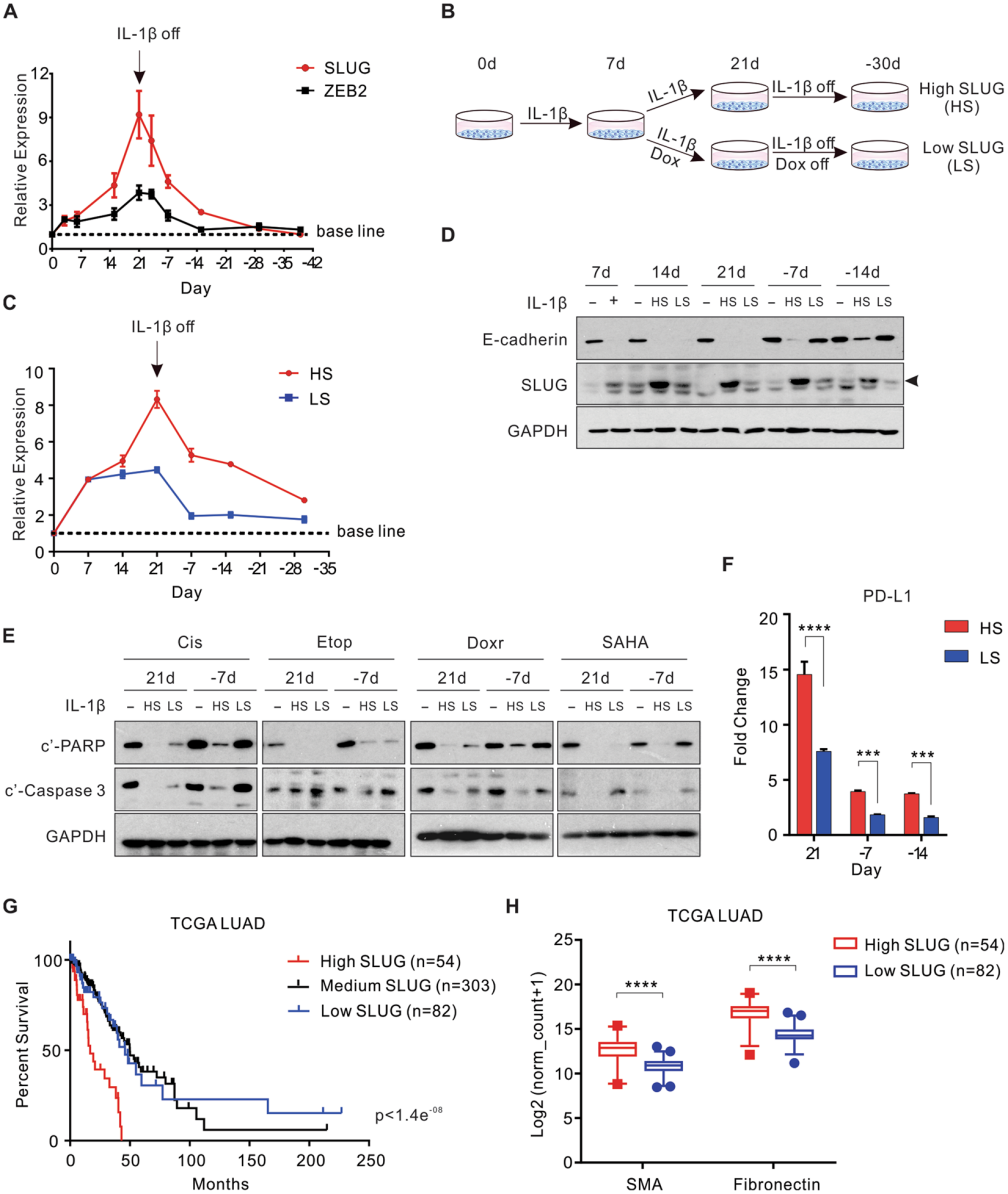
Accumulation of SLUG is indispensable for the establishment of the memory phenotypes. (**A**) A crescendo expression pattern of SLUG and ZEB2 upon the chronic IL-1β exposure in A549 cells, followed by a decrescendo pattern after IL-1β withdrawal, determined by RT-qPCR. (**B**) Experimental design: in LS group, doxycycline was added to induce SLUG shRNA after 7 days of IL-1β treatment and removed together with IL-1β at day 21. (**C**) Relative SLUG expression by RT-qPCR in LS and HS cells compared to the untreated cells. (**D**–**F**) EMT markers, apoptosis resistance, and surface PD-L1 expression were evaluated in different EMT stages in LS and HS cells compared to the IL-1β-untreated cells. (**G**) Kaplan-Meier curves from the TCGA lung adenocarcinoma (LUAD) cohort showing the survival probability in patients with various levels of SLUG expression. (**H**) Expression of SMA and Fibronectin in the same cohort of patients (95% confidence interval). All results are reported as mean ± SEM unless indicated otherwise. ***P < 0.0002, ****P < 0.0001. See also Fig. S3.

Following the acute IL-1β exposure, RNA sequencing revealed upregulation of two EMT transcription repressors, *SLUG* and *ZEB2* (Table S1). RT-PCR confirmed that the expression of SLUG and ZEB2, but not SNAI1, ZEB1, or TWIST1, increased in A549 cells (Fig. S3A). Of mechanistic importance, knockdown of SLUG or ZEB2 was sufficient to abolish the downregulation of E-cadherin in acute EMT (Fig. S3B). In chronic EMT, SLUG expression dramatically increased with continous IL-1β exposure (up to 9-fold) whereas *ZEB2* was only upregulated less than 4-fold (Fig. 3A). Therefore, we hypothesized that the elevated SLUG expression was essential for the establishment of chronic EMT and EMT memory. To test this hypothesis, we generated A549 cells expressing doxycycline (Dox)-inducible SLUG shRNA and partially knocked down SLUG after an initial 7-day IL-1β exposure by addition of Dox from day 8 to day 21 (Fig. 3B). These cells were designated as “low SLUG” (LS), compared to cells maintaining the endogenous levels of SLUG without knockdown, designated as “high SLUG” (HS). Whereas HS cells demonstrated a steadily increased SLUG expression up to 21 days of IL-1β exposure, this upregulation was abolished after 7 days of IL-1β exposure in LS cells. Both SLUG mRNA and protein levels in LS cells reversed rapidly to the base line after IL-1β withdrawal (Fig. 3C,D). Consistently, LS cells displayed a less profound mesenchymal morphology in chronic EMT compared to HS cells, and readily reversed to the epithelial features (Fig. S3C). A complete reversal of E-cadherin expression in LS cells at day 7 of IL-1β withdrawal confirmed an impaired EMT memory phenotype (Fig. 3D). In addition, apoptosis resistance and elevated PD-L1 expression were also readily reversed in LS cells (Fig. 3E,F). These data indicate that IL-1β-induced high SLUG expression is indispensable for the establishment of chronic EMT and EMT memory.

To assess whether high SLUG level was sufficient to induce these memory phenotypes, we generated A549 cells overexpressing various levels of SLUG controlled by Dox. Cells highly overexpressing SLUG harbored the most profound EMT phenotype, highest apoptosis resistance, and highest PD-L1 expression. These phenotypes were memorized for at least 7 days following the termination of SLUG overexpression. In contrast, cells with moderate SLUG expression demonstrated moderate EMT and impaired memory phenotypes (Fig. S3D–G). These findings suggest that SLUG alone can establish both EMT and EMT memory phenotypes in a concentration-dependent manner. To investigate the potential clinical relevance of these findings, we performed Kaplan-Meier analysis using the TCGA database and found that high SLUG expression in lung adenocarcinoma patients was associated with poor prognosis (Fig. 3G). Two mesenchymal markers, SMA and Fibronectin, were also more highly expressed in these patients with high SLUG expression tumors (Fig. 3H).

### Pathways mediating acute EMT are not required for the maintenance of chronic EMT or EMT memory

**Figure 4:**
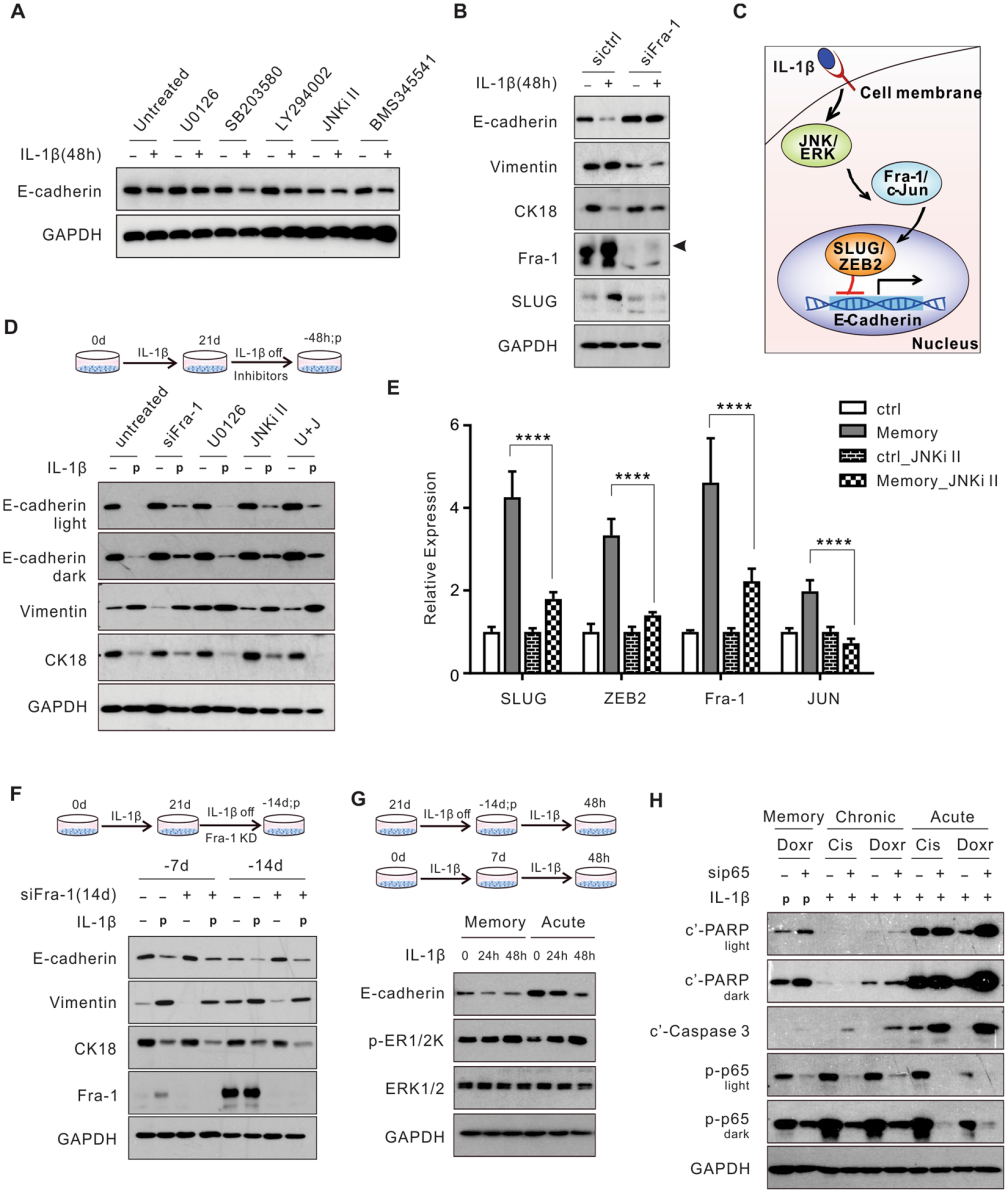
Pathways mediating acute EMT are not required for the maintenance of chronic EMT or EMT memory. (**A**) E-cadherin expression following 48 hours of chemical inhibition of IL-1β-activated signaling pathways. (**B**) A549 cells were exposed to IL-1β for 48 hours following Fra-1 knockdown and examined for EMT markers. (**C**) A schematic mechanism of the IL-1β-induced acute EMT in A549 cells. (**D**) Evaluation of EMT markers with 48-hour inhibition of the MAPK (ERK and JNK) - AP-1 - SLUG axis in EMT memory. (**E**) Expression of SLUG, ZEB2, Fra-1, and JUN upon the JNK inhibition in EMT memory (-7d). **(F**) Expression of EMT markers with 14 days of Fra-1 knockdown (KD) following IL-1β withdrawal from the chronic treatment. (**G**) E-cadherin level and ERK signaling activity in A549 cells when being re-exposed to IL-1β after either 14 days of IL-1β withdrawal in EMT memory or 7 days of IL-1β exposure in acute EMT. (**H**) Evaluation of chemotherapy-induced apoptosis upon p65 knockdown in acute (48 hours), chronic EMT (24 days), and EMT memory (-7 days). U0126, ERK pathway inhibitor; SB203580, p38 inhibitor; LY294002, PI3K inhibitor; JNKi II, JNK inhibitor; BMS345541, IKK inhibitor; “U + J”, U0126 and JNKi II combination treatment. “-d/-h”, days/hours after IL-1β withdrawal; “p”, previously treated with IL-1β. Cis, cisplatin; Doxr, doxorubicin. All results are reported as mean ± SEM. ****P < 0.0001. See also Fig. S4.

Next, we sought to delineate the molecular events upstream of SLUG in the regulation of IL-1β-induced EMT in A549 cells. IL-1β activated the MAPK (ERK, JNK, and p38), AKT, and NF-κB signaling pathways within 48 hours of treatment (Fig. S4A). Reagents inhibiting the ERK or JNK pathway, impaired the downregulation of E-cadherin in acute EMT (Fig. 4A). This inhibition was concentration-dependent (Fig. S4B). Among the downstream targets of the ERK and JNK pathways, we found that Fra-1 and c-JUN, were phosphorylated and upregulated in response to IL-1β, leading to the formation of the transcription factor AP-1 by dimerization (Fig. S4C,D). Transient knockdown of Fra-1 prevented the upregulation of SLUG and subsequent acute EMT (Fig. 4B). The JNK inhibitor II abolished the upregulation of Fra-1, SLUG, and ZEB2 (Fig. S4E). These data suggest activation of the JNK - AP-1 - SLUG signaling pathway following the acute IL-1β exposure. Thus, we have identified that the MAPK (ERK and JNK) - AP-1 - SLUG axis mediates the IL-1β-induced acute EMT in A549 cells (Fig. 4C).

Because SLUG expression and ERK phosphorylation remained elevated in both chronic EMT and EMT memory compared to the untreated cells (Figs. 3A and S4F), we anticipated that the MAPK (ERK and JNK) - AP-1 - SLUG axis was also required to maintain chronic EMT and EMT memory. Therefore, we hypothesized that inhibition of this axis should reverse the mesenchymal features in the EMT memory cells. However, genetic or chemical inhibition of the MAPK (ERK and JNK) - AP-1 - SLUG axis for 48 hours in EMT memory failed to reverse the expression of the EMT markers (Fig. 4D,E). Furthermore, extension of Fra-1 knockdown for 14 days in EMT memory did not alter the expression of the EMT markers (Fig. 4F). Similar findings were found in cells with chronic EMT (Fig. S4G). These results indicate that the MAPK (ERK and JNK) - AP-1 - SLUG axis is not required for E-cadherin suppression in either chronic EMT or EMT memory once the suppression is established, consistent with the findings that EMT phenotypes are maintained even after the SLUG overexpression is terminated (Fig. S3D–G). Moreover, we re-exposed cells to IL-1β in EMT memory to determine if this re-stimulation would restore the molecular features of EMT. In contrast to the cells in acute EMT, IL-1β re-exposure failed to further decrease E-cadherin in cells with EMT memory despite activation of ERK signaling, indicating that E-cadherin suppression is memorized and cannot be altered by additional stimulation (Fig. 4G). Similarly, we determined that the NF-κB inhibition by p65 knockdown strongly sensitized cells to apoptosis in response to cisplatin and doxorubicin in acute EMT. However, this inhibition achieved minimal “rescue” effects in chronic EMT and EMT memory (Fig. 4H). These results suggest that the NF-κB pathway is more critical for apoptosis resistance in acute EMT than chronic EMT and EMT memory. Together, these findings indicate that the pathways mediating the acute EMT phenotypes are not necessarily required to maintain the chronic EMT and EMT memory phenotypes.

### Dynamic epigenetic modifications of the *CDH1* promoter in IL-1β-induced EMT

Epigenetic modifications can regulate epithelial gene expression during EMT, leading to either long-term silencing or disinhibition of these genes during MET^28^. We found that once established, IL-1β-induced EMT memory was maintained independent of the MAPK (ERK and JNK) - AP-1 - SLUG axis. Therefore, we hypothesized that epigenetic machinery, including histone modifications and DNA methylation, may mediate EMT memory. Because downregulation of E-cadherin (*CDH1*) is a direct function of SLUG and a hallmark of EMT, we focused on investigating the epigenetic modifications of the *CDH1* promoter in A549 cells.

**Figure 5:**
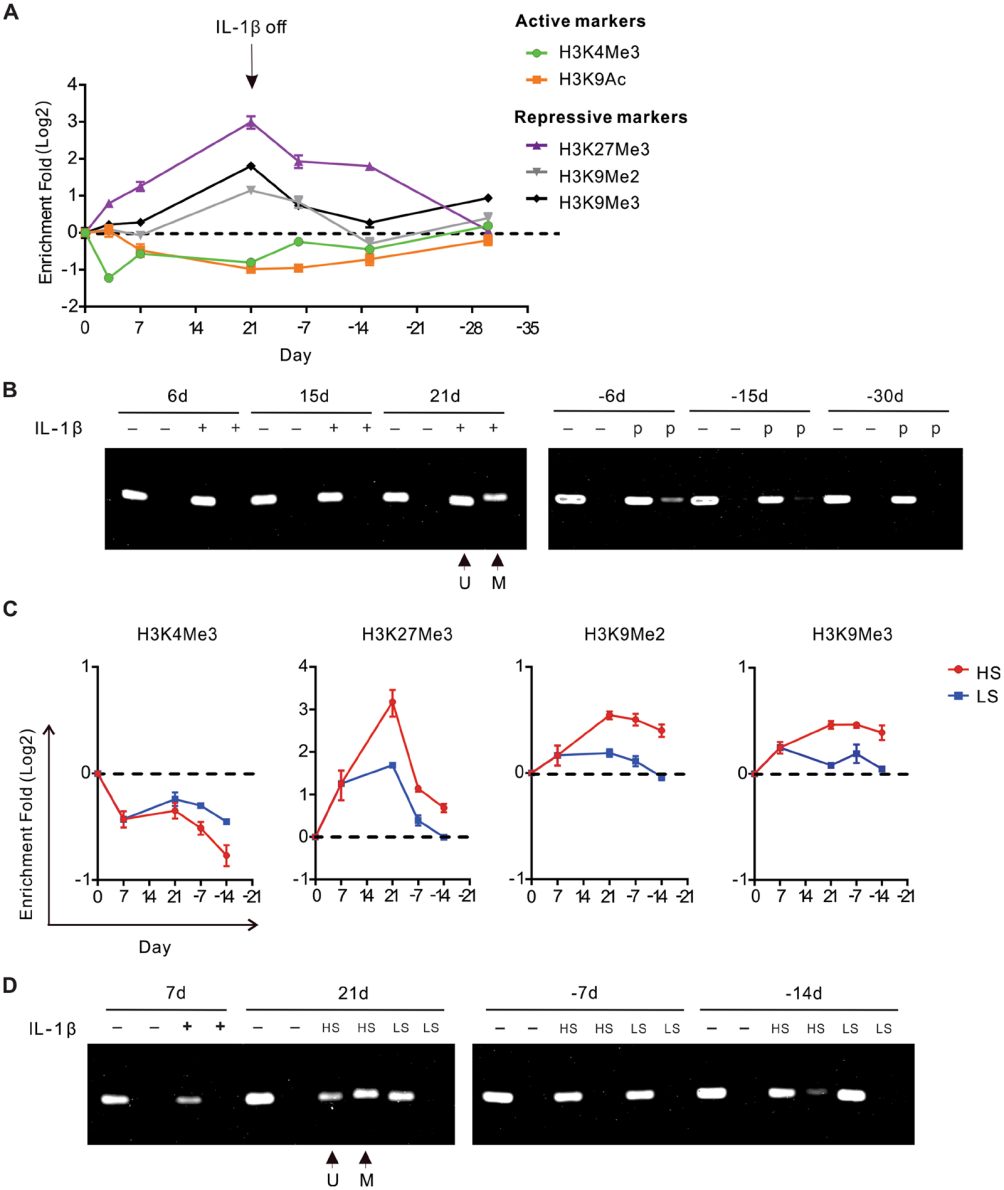
Dynamic epigenetic modifications of the *CDH1* promoter in IL-1β-induced EMT. **(A**) Relative fold changes of histone modifications in different EMT stages compared to the untreated cells by ChIP-qPCR. (**B**) A representative picture of DNA electrophoresis showing the relative abundance of methylated (M, higher band) and unmethylated (U, lower band) DNA, examined by MSP. DNA methylation level was determined by the ratio of M band to U band in each sample. (**C**) SLUG level-dependent histone modifications compared to the untreated cells by ChIP-qPCR. (**D**) DNA methylation upon IL-1β exposure in HS and LS cells, examined by MSP. Values from ChIP-qPCR were in log scale and normalized to IgG control and Tubulin control at each time point. “-d”, days after IL-1β withdrawal; “p”, previously treated with IL-1β. See also Fig. S5.

To characterize histone modifications, we performed ChIP-qPCR and revealed temporal epigenetic alterations of the *CDH1* promoter. Upon the acute IL-1β exposure, there was a reduction of active histone modifications including H3K9 acetylation (H3K9Ac) and H3K4 trimethylation (H3K4Me3), and an enrichment of the repressive histone modification H3K27 trimethylation (H3K27Me3). These epigenetic alterations were enhanced in the continuous presence of IL-1β, persisted after IL-1β withdrawal, and eventually returned to basal levels, correlating with E-cadherin expression (Fig. 5A). Notably, two repressive histone modifications, H3K9 dimethylation (H3K9Me2) and H3K9 trimethylation (H3K9Me3), were only enriched in chronic EMT and in the early phase of EMT memory (Fig. 5A). Because H3K9Me2 and H3K9Me3 often co-existed with DNA methylation, we further examined DNA methylation by methylation-specific PCR (MSP) and found increased methylated DNA in chronic EMT and EMT memory but not in acute EMT (Fig. 5B). Consistently with the memorized E-cadherin suppression, chronic TNF-α and TGF-β exposure also led to increased DNA methylation in the *CDH1* promoter (Fig. S5A).

Next, we investigated the mechanism underlying these dynamic epigenetic modifications. Studies by Javaid *et al*. showed dynamic chromatin modifications in a system of Snail overexpression-induced EMT^32^. Others also reported that Snail could directly interact with histone methyltransferases such as G9a and SUV39H1 to suppress E-cadherin in breast cancer^33,34^. Because we found that the highly elevated SLUG level was essential for the establishment of chronic EMT and EMT memory, we postulated that SLUG mediated these epigenetic alterations upon IL-1β exposure. Utilizing the cells with partial SLUG knockdown, we found that compared to HS cells, LS cells contained more active histone modifications, such as H3K4Me3, and fewer repressive histone modifications, such as H3K27Me3 and H3K9Me2/3 in the *CDH1* promoter (Fig. 5C). In addition, there was no increase of DNA methylation in LS cells despite the chronic IL-1β exposure (Fig. 5D). Next, we assessed the expression of the enzymes involved in these histone modifications as well as DNA methylation following IL-1β exposure but could not identify any targets (Fig. S5B). Although increased HDAC9 expression correlated well with decreased H3K9Ac, knockdown of HDAC9 failed to reverse E-cadherin expression, possibly due to the redundancy of HDACs (Fig. S5C). These findings demonstrate that H3K4 and H3K27 trimethylation as well as H3K9Ac are the immediate responders to IL-1β exposure, while H3K9 and DNA methylation only occurs following chronic IL-1β exposure. In addition, these epigenetic alterations in the *CDH1* promoter are SLUG-dependent.

### Blockade of epigenetic modifications restores E-cadherin expression and cellular sensitivity to chemotherapy in EMT memory

**Figure 6:**
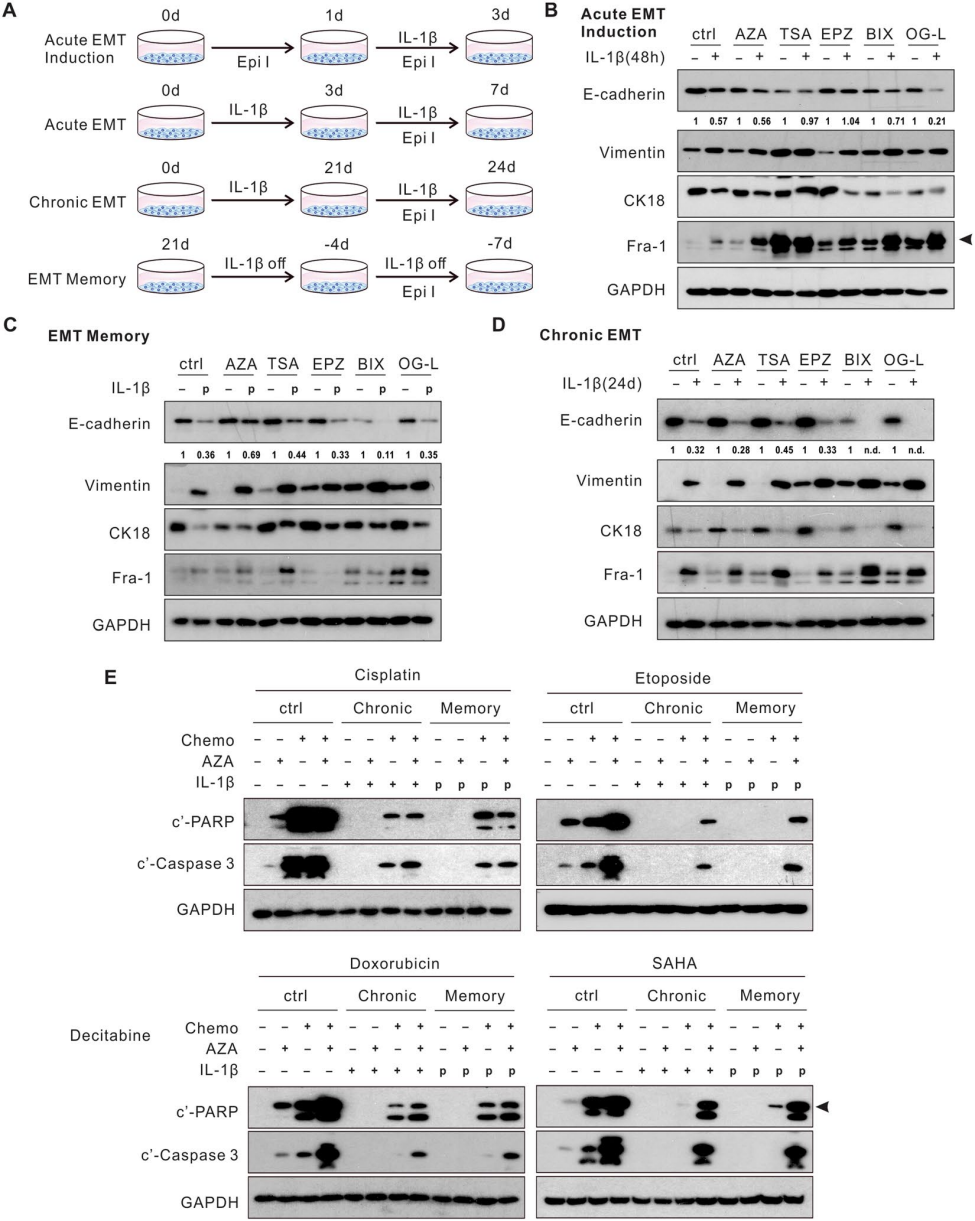
Blockade of epigenetic modifications restores E-cadherin expression and cellular sensitivity to chemotherapy in EMT memory. (**A**) A schematic diagram of the experimental design. (**B**) Pre-treatment of TSA and EPZ prevented E-cadherin downregulation upon the acute IL-1β exposure. (**C**) AZA treatment restored E-cadherin expression in EMT memory. (**D**) Epigenetic inhibitors did not alter E-cadherin expression in chronic EMT. (**E**) Apoptosis markers were evaluated in chronic EMT and EMT memory following the combination of AZA and chemotherapy agents. Quantification of E-cadherin expression was normalized to samples without IL-1β exposure. Epi I, epigenetic inhibitors. AZA, 5′-azacytidine-2′-deoxycytidine (decitabine), DNA methyltransferase inhibitor; TSA, pan HDAC inhibitor; EPZ, EPZ-6438, EZH2 inhibitor; BIX, BIX01294, G9a inhibitor; OG-L, OG-L002, LSD1 inhibitor. “p”, previously treated with IL-1β. “n.d.”, non-detectable. See also Fig. S6.

To determine the significance of these epigenetic modifications in the different stages of EMT, we chemically inhibited the relevant enzymes (Fig. 6A). Both the histone acetylation inhibitor (HDACi), Trichostatin A (TSA), and the H3K27Me3 inhibitor, EPZ-6438, blocked the downregulation of E-cadherin following the acute IL-1β exposure (Figs. 6B and S6A). In contrast, neither TSA nor EPZ-6438 reversed E-cadherin expression in chronic EMT and EMT memory (Fig. 6C,D). Consistent with these findings, knockdown of EZH2, the enzyme predominantly responsible for H3K27Me3, impaired E-cadherin downregulation in acute EMT, but not in chronic EMT or EMT memory (Fig. S6B). A DNA methyltransferase inhibitor (DNMTi), 5-azacytidine-2′-deoxycytidine (decitabine), reversed E-cadherin expression in EMT memory (Fig. 6C). Inhibition of H3K9Me2/3 either by BIX01294 or genetic knockdown of SETDB1 or SUV39H1, two enzymes engaged in H3K9Me2/3, failed to reverse E-cadherin expression in EMT memory or chronic EMT (Figs. 6C,D and S6C). Of note, none of the chemical inhibitors were able to impair E-cadherin suppression in chronic EMT (Fig. 6D). These results indicate that following the IL-1β exposure, H3K27Me3 and H3K9Ac mediate the downregulation of E-cadherin in acute EMT while DNA methylation is responsible for the E-cadherin suppression in EMT memory.

Next, we examined whether these epigenetic inhibitors could enhance chemotherapy-induced apoptosis in cells with EMT memory. Decitabine enhanced apoptosis induced by Etoposide, Doxorubicin, and SAHA not only in the epithelial cells, but also in the cells with chronic EMT and EMT memory. Notably, combination of decitabine with SAHA induced a comparable level of apoptosis in both the epithelial and mesenchymal cells **(**Fig. 6E). However, in combination with Cisplatin, decitabine had minimal effects, consistent with previous clinical findings^35^. Together, these findings suggest that E-cadherin suppression in the different stages of IL-1β-induced EMT is driven by distinct epigenetic modifications. Inhibition of DNA methylation not only reverses E-cadherin expression but also increases the susceptibility to chemotherapy in these mesenchymal cells.

**Figure 7:**
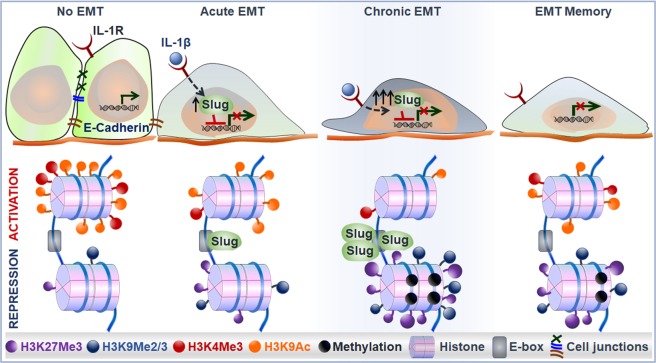
A schematic model of SLUG-dependent epigenetic modifications of the *CDH1* promoter following IL-1β exposure in A549 cells.

Overall, we have found SLUG-dependent epigenetic modifications of the *CDH1* promoter following IL-1β-induced EMT. Upon acute IL-1β exposure, upregulated SLUG reduces the activating histone modifications, including H3K4Me3 and H3K9Ac, and promotes H3K27Me3, a suppressive histone modification. In the continuous presence of IL-1β, SLUG accumulation leads to further enrichment of H3K27Me3 and *de novo* H3K9Me2/3 as well as DNA methylation, which contributes to memorized E-cadherin suppression in EMT memory (Fig. 7).

## Discussion

The elevated incidence of cancer and resulting mortality in patients with chronic inflammatory conditions highlight the significance of chronic inflammation in tumor initiation, progression, and metastasis. Preexisting chronic inflammation can predispose normal cells to environmental carcinogens by disrupting the biological barriers of normal tissue and also activate the intrinsic epigenetic machinery in epithelial cells, resulting in activation of oncogenes and/or inactivation of tumor suppressors^2,36^. Both precancerous and tumor cells can exploit deregulated cytokines in the TME, promoting growth through activation of downstream signaling, such as the ERK, JUN, and NF-κB pathways. These inflammatory pathways further promote cancer cell stemness, enhance resistance to environmental or therapeutic pressure, and facilitate metastasis^18,37^. In addition, immune cells in the TME are highly plastic and under the influence of inflammatory mediators, can be redirected to exert pro-tumor functions through multiple mechanisms, including induction of DNA damage by producing reactive oxygen species, suppression of immune responses by upregulating checkpoint molecules, and precipitation of metastasis by forming complexes with cancer cells^36,38,39^.

In this study, we found gradually progressing EMT phenotypes upon chronic cytokine exposure, followed by an EMT memory phenomenon in a subset of NSCLC cell lines. We observed that accumulation of the EMT transcription factor SLUG was essential for the establishment of both chronic EMT and EMT memory. This molecular mechanism downregulating E-cadherin in acute EMT was not required for maintaining E-cadherin suppression in chronic EMT or EMT memory. The molecular difference was found to be attributed to dynamic epigenetic modifications. Inhibition of DNA methylation elevated E-cadherin expression during the EMT memory phase and enhanced sensitivity to chemotherapy agent-induced apoptosis in both chronic EMT and EMT memory.

Because transcriptional factors, such as SLUG and Fra-1, were required in acute EMT, it was anticipated that they also would be indispensable in chronic EMT and EMT memory. However, the maintenance of E-cadherin suppression in EMT memory relied on histone modifications and DNA methylation, independent of SLUG expression. Similarly, the NF-κB pathway, which mediated apoptosis resistance upon the acute IL-1β exposure, appeared not to be critical in chronic EMT and EMT memory. These findings suggest that inflammatory pathways regulate target gene expression and modulate tumor cell EMT in a complex temporal manner.

Whereas SLUG appears to be a major mediator of the epigenetic control of E-cadherin expression, the interactions among different epigenetic modifications may also be important^40,41^. For example, H3K9 methylation and DNA methylation are considered to be highly interrelated, as evidenced by their co-existence in heterochromatin and the direct interactions between H3K9 and DNA methyltransferases^42,43^. Several studies have provided evidence of the association between H3K27Me3 and DNA methylation: (i) promoters marked with H3K27Me3 are able to gain *de novo* DNA methylation during embryogenesis and carcinogenesis^44,45^; (ii) biochemical studies demonstrated binding of DNMTs to EZH2^46^; and (iii) co-occurrence and interaction between H3K27 and H3K9 methylation^47,48^. Concordantly, we have demonstrated that methylation of both H3K9 and DNA is elevated following the enrichment of H3K27Me3 upon chronic IL-1β exposure. Therefore, it is possible that the accumulation of H3K27 contributes to the *de novo* H3K9 and DNA methylation. Genetic or chemical inhibition of H3K27Me3 could restore E-cadherin expression only in acute EMT but not in chronic EMT or EMT memory. These findings suggest a hierarchy of epigenetic events leading to E-cadherin silencing in EMT.

Given that the epigenetic regulation upon chronic IL-1β exposure is SLUG-level dependent, the decrease in H3K27Me3, H3K9Me2/3, and DNA methylation following IL-1β withdrawal is most likely due to inadequate SLUG levels, leading to gradual restoration of E-cadherin expression. This could be attributed to disappearance of the previously formed epigenetic modifications. It is also possible that these previously formed epigenetic modifications are diluted upon cell division because studies have shown that the *de novo* formation of epigenetic modifications can, in certain instances, be permanent. Bintu *et al*. demonstrated a dynamic epigenetic regulation in gene suppression at a single-cell level utilizing time-lapse microscopy^49^. The authors found that arbitrary expression of embryonic ectoderm development (EED), a structural component functioning with EZH2, was sufficient to induce irreversible gene silencing. Higher expression of EED resulted in enrichment of H3K27Me3 and H3K9Me3 and endowed more cells with permanent gene silencing. Future studies tracing these epigenetic alterations at single cell resolution in this inflammation-induced EMT model will address this possibility and further dissect the composition of the epigenetic machinery in maintaining gene silencing.

Although it is well known that EMT endows cells with metastatic capacity, analysis of tissue specimens from metastatic tumors often reveals cells with epithelial features. EMT plasticity therefore is proposed to temporally modify these properties by facilitating cellular responses to the microenvironmental stimuli that lead to mesenchymal phenotypes and metastatic behaviors^23,24,27^. In the current study, fading of EMT memory, accompanied by a gradual elevation of E-cadherin expression, is consistent with a profound EMT plasticity. In a case of acquired EMT, increased migration and invasion of tumor cells enable them to travel away from primary tumor sites, which also distance them from EMT-promoting stimuli, such as inflammatory factors in the primary TME. We propose that because of the memorized EMT phenotypes, these migratory cells are able to seed the metastatic spread to distant organ sites. Once colonizing at a distant organ, the metastatic cells require restoration of the epithelial phenotypes in order to establish macrometastasis. Thus, chronic inflammation-induced EMT memory may be responsible for the underlying mechanism enabling spatial-temporal EMT plasticity associated with metastatic competence.

In addition to the anti-proliferative effects of DNMTi and HDACi, we have found that a low dose of DNMTi combined with an HDACi induces significant apoptosis in cells with chronic EMT and EMT memory. A phase I/II clinical trial evaluating a combination of a low dose of the DNMTi decitabine and the HDACi entinostat demonstrated durable responses lasting more than one year in some patients with recurrent metastatic NSCLC^50^. There are also ongoing investigations assessing the efficacy of combining epigenetic therapies with other cancer treatments, such as chemotherapy or immunotherapy. In the above-mentioned trial, five patients were subsequently enrolled in anti-PD-1/PD-L1 therapies due to disease progression. All five patients achieved apparent clinical benefit^50–52^. Consistent with these findings, Topper *et al*. recently found that DNMTi and HDACi combination therapy induced potent immune responses, including an enhanced interferon gamma signature, CD8 T cell infiltration, and PD-L1 expression in a murine NSCLC model, supporting the potential benefit of checkpoint inhibitor therapy following epigenetic therapies^53^. Larger clinical trials are underway, directly comparing the efficacy of the combination therapy to immunotherapy alone (NCT01935947, NCT02546986, NCT02437136).

Inflammation profoundly impacts tumor development, progression, and metastasis. Our current findings are consistent with the remarkable reduction of both lung cancer incidence (67%) and mortality (77%) demonstrated by IL-1β inhibition in the CANTOS trial^13^. Our studies amplify mechanistic insights implicating IL-1β-regulated chronic inflammation as a central component in carcinogenesis and metastasis. Thus, inhibition of this pathway may contribute to both prevention and treatment of NSCLC.

## Materials and Methods

### Cell lines and reagents

NSCLC cell lines A549, H460, and A427 were purchased from ATCC and cultured as previously described^14^. Cells with doxycycline-inducible shRNA were constructed as previously described^54^. Briefly, the *SLUG* shRNA constructs were recombined into pLentipuro3/BLOCK-iT-DEST. The resulting recombinant lentiviruses were produced by 293 T cells and transduced into cells with the Tet repressor protein from the pLenti0.3/EF/GW/IVS-Kozak-TetR-P2A-Bsd vector. Cells were selected in 2 µg/mL puromycin for 10 days. Cells with doxycycline-controlled SLUG overexpression were made by transducing the pLVX-Tet-off Advanced vector and Plvx-Tight-Puro vector (Clontech) and selected in 2 µg/mL puromycin for 10 days. All cell lines were routinely genotyped and tested for mycoplasma. Recombinant IL-1β was purchased from BD Pharmingen and used at 1 ng/ml unless stated otherwise. For chronic IL-1β treatment, cells were split and passaged every 3 to 4 days with fresh IL-1β added to the medium.

### Chemical treatment

For assessment of apoptosis resistance, cells were treated with either Cisplatin (50 µM), Etoposide (100 µM), Doxorubicin (20 µM), or SAHA (20 µM) for 24 hours. For pathway and epigenetic inhibition, cells were treated with inhibitors for 72 hours at the following concentrations: 50 nM for TSA, 1 uM for BMS345541 and BIX01294, 5 uM for U0126, SB203580, and 5-aza-2′-deoxycytidine, 10 uM for JNK inhibitor II and LY294002, 20 uM for EPZ-6438, and 50 uM for OG-L002. U0126 was purchased from Cell Signaling Technology; NF-κB inhibitor BMS345541, histone deacetylase inhibitor Trichostatin A (TSA), and DNA methylation inhibitor 5-aza-2′-deoxycytidine (decitabine) from Sigma; AKT inhibitor LY294002, JNK inhibitor II, and p38 inhibitor SB203580 (SB) from Calbiochem; LSD1 inhibitor OG-L002, G9a inhibitor BIX01294, EZH2 inhibitor EPZ-6438, and all four chemotherapy reagents were purchased from Selleckchem.

### Transient transfection of siRNA

Cells were transfected in a 6-well plate with siRNA using Lipofectamine RNAiMAX (Life Technologies) at a final concentration of 15 nmol/L following overnight attachment. For transient gene repression, cells were cultured continuously for 72 hours with the proper treatments. For prolonged gene repression, cells were repeatedly transfected with siRNA every 4 days. SiRNA pools against *SLUG*, *ZEB2* and *Fra-1* were purchased from Dharmacon.

### Western blot

Samples were prepared as previously stated^55^. Horseradish peroxidase-conjugated secondary antibodies (Bio-Rad, Hercules, CA) and enhanced chemiluminescence (ECL) reagent (Amersham Biosciences, Piscataway, NJ) were used for protein detection. Antibodies against phosphorylated-p65, p-MAPK, p-JNK(T183/Y185), p-c-JUN(S73), c-JUN, p-p38, p-Fra-1, Fra-1, SLUG, β-actin, and α-tubulin were purchased from Cell Signaling Technology. Antibodies against E-cadherin and Vimentin were purchased from BD Pharmingen. The antibodies against cytokeratin 18 (CK18) and GAPDH were from Abcam and Advanced Immunochemical Inc, respectively.

### Quantitative reverse transcription PCR

Total RNA and cDNA were prepared as previously stated^56^. Transcript levels were measured using a MyiQ Cycler (Bio-Rad). Taqman Probes for *SNAI1, SLUG, ZEB1, ZEB2, TWIST1, CJUN, JUNB, CFOS, FRA1(FOSL1), FOSL2*, and *B2M* were purchased from Life Technologies. Primers for *DNMT1, DNMT2, DNMT3, EZH2, EHMT1, EHMT2, SETDB1, SUV39H, HDAC1, HDAC2, HDAC3, HDAC4, HDAC6, HDAC9*, and *GAPDH* were obtained from PrimerBank and utilized with Cyber-Green-based qPCR system. Amplification was carried out for 40 cycles of 15 seconds at 95 °C, 30 seconds at 55 °C and 30 seconds at 72 °C. All samples were run in triplicate, and the relative gene expression levels were determined by normalizing their expression to *B2M* or *GAPDH*. Expression data are presented as fold-change values relative to the normalized expression levels in a reference sample using the following equation: RQ = 2^−ΔΔCt^.

### Proliferation assay

As an indication of cell viability and proliferation, cellular ATP levels were measured using the ATPlite 1step Luminescence Assay Kit (Perkin Elmer). A549 cells with the indicated IL-1β treatment were plated in 96-well plates at 1000 cells per well. Eight replicates for each condition were plated for each independent experiment. ATP luminescence was assessed every 24 hours up to 72 hours. Readings at each time point were normalized to the reading obtained at baseline to control for plating differences.

### AIG assay

A modified high-throughput cell transformation assay was utilized to evaluate soft agar colony growth as previously described^56^. Briefly, the cells were suspended in 0.4% agar and plated atop a thick layer of solidified 0.6% agar. A549 cells with the indicated IL-1β treatment were plated in 96-well plates at 750 cells per well. Cells were cultured in complete medium for a total of 14 days. Data were collected as previously described^56^. At least ten replicates for each condition were plated for each independent experiment.

### Flow cytometry

Cells were trypsinized and washed with FACS buffer as previously indicated. PD-L1 antibody (BD Bioscience) was diluted at 1:100 and stained cells for 30 mins at RT. Cells were washed twice before analysis by flow cytometry (BD, LSRII).

### ChIP-qPCR

ChIP assays were performed based on the manufacturer’s protocol (Millipore). Briefly, 5 million cells were collected and cross-linked with 1% of formaldehyde at RT, followed by cell nucleus extraction. The cell lysate was then subjected to sonication and then incubated with 1ug of either IgG or the antibody of interest overnight in the presence of magnetic protein A/G beads. Bound DNA-protein complexes were eluted and reverse-cross-linked after a series of washes. Quantitative-PCR assays were performed under standard conditions. The primers for the E-cadherin promoter were 5′- CCACGCACCCCCTCTCAGT -3′and 5′- GAGCGGGCTGGAGTCTGAAC -3′; for the negative control, the loci were 5′- TCTTGACCTCTCCGCATC -3′ and 5′-CAACAGGACGAATGTGACTG -3′.

### Methylation specific PCR (MSP)

MSP was performed using bisulfate-modified DNA under conditions that have been previously described^57^. Briefly, genome DNA was extracted using the Genome DNA Extraction Kit (Zymo Research) and subjected to bisulfate conversion following the manufacturer’s protocol. The primers for the E-cadherin promoter were: 5′-TTAGGTTAGAGGGTTATCGCGT-3′ and 5′-TAACTAAAAATTCACCTACCGAC-3′ for methylated DNA; 5′-TAATTTTAGGTTAGAGGGTTATTGT -3′and 5′-CACAACCAATCAACAACACA-3′ for non-methylated DNA. The PCR products were then electrophoresed on 2% agarose gels, stained with ethidium bromide, and visualized under UV illumination.

### RNA sequencing and differential expressed genes

Cell lysates were harvested using 400 μLQiazole (Qiagen), and total RNA was extracted according to the manufacturer’s protocol (Zymo Research). Library was prepared and sequenced by Illumina Hiseq. 3000 at the UCLA Technology Center for Genomics and Bioinformatics. Single-end transcriptome reads were mapped to the Ensemble GRCh37 reference genome using *Tophat2*. *HTSeq-count* was used to count the reads for each gene^58^. *EdgeR* was then utilized to normalizeand identify differentially expressed genes based on negative binomial distribution. A gene was defined as differentially expressed between two conditions if i) its Benjamini and Hochberg based false discovery rate was less than 0.05, and ii) its fold change was more than 2. Average log-ratio expressions of identified differential expressed genes were then subjected to hierarchical cluster analysis by utilizing Cluster 3.0 with metric based on cosine similarity and average linkage in clustering approach. The sequencing data is accessible at GEO (GSE142620).

### Pathway analysis

Gene Set Variant Analysis (GSVA) was utilized to derive the activity of interest pathway or gene set from the expression of involved genes at various time points^59^. The derived dynamic profile of pathway activities was then subjected to the time-course analysis by utilizing R package *maSigPro* to determine if temporal profiles were statistically significant between the treatment and control conditions^60^. In brief, the analysis performed a two-step regression approach to determine the pathways whose activities were time dependent, and significant different between treatment and control groups. A pathway was identified being deregulated in the experimental compared to the control groups if (i) R-squared of the final regression model for its activities was >0.7, and (ii) Benjamini and Hochberg based false discover rates of the regression coefficients associated experimental group were <1e^−5^. Furthermore, deregulated pathways were stratified into memory exhibiting group if its activities in experimental compared to the control groups remained more than 50% of their peak values 15 days after IL-1β withdrawal, otherwise they were defined as IL-1β sensitivity. Information regarding the gene members of total 10,611 gene sets was obtained from the Molecular signature database^31^.

### TCGA lung adenocarcinoma data

RSEM normalized expression (level 2) and clinical information was downloaded from the data portal. *SLUG* expression levels were transformed to z-scores based on those observed in normal samples. Samples were categorized in three groups as high (z-score > 2), intermediate (−2 ≤ z-score ≤ 2) and low (z-score < −2) groups.

### Statistical analysis

All statistical analyses were performed in Prism 6 (GraphPad, La Jolla, CA) unless otherwise noted. Student two tailed t-test was utilized to determine the statistical significance between experimental conditions with the exception of those in our RNA sequencing analysis which is described above.

## Supplementary information

Dataset 1.

Dataset 2.

## References

[1] Coussens LM, Werb Z (2002). Inflammation and cancer. Nature.

[2] Grivennikov SI, Greten FR, Karin M (2010). Immunity, inflammation, and cancer. Cell.

[3] Hanahan D, Weinberg RA (2011). Hallmarks of cancer: the next generation. Cell.

[4] Houghton AM (2013). Mechanistic links between COPD and lung cancer. Nat Rev Cancer.

[5] Wilson DO (2008). Association of radiographic emphysema and airflow obstruction with lung cancer. Am J Respir Crit Care Med.

[6] Collins RH, Feldman M, Fordtran JS (1987). Colon cancer, dysplasia, and surveillance in patients with ulcerative colitis. A critical review. N Engl J Med.

[7] Rothwell PM (2011). Effect of daily aspirin on long-term risk of death due to cancer: analysis of individual patient data from randomised trials. Lancet.

[8] Cuzick J (2009). Aspirin and non-steroidal anti-inflammatory drugs for cancer prevention: an international consensus statement. Lancet Oncol.

[9] Wu C (2016). Correlation between serum IL-1beta and miR-144-3p as well as their prognostic values in LUAD and LUSC patients. Oncotarget.

[10] McLoed AG (2016). Neutrophil-Derived IL-1beta Impairs the Efficacy of NF-kappaB Inhibitors against Lung Cancer. Cell Rep.

[11] Millares L (2018). Tumor-associated metabolic and inflammatory responses in early stage non-small cell lung cancer: Local patterns and prognostic significance. Lung Cancer.

[12] Shiels MS (2013). Circulating inflammation markers and prospective risk for lung cancer. J Natl Cancer Inst.

[13] Ridker PM (2017). Effect of interleukin-1beta inhibition with canakinumab on incident lung cancer in patients with atherosclerosis: exploratory results from a randomised, double-blind, placebo-controlled trial. Lancet.

[14] Krysan K (2005). Prostaglandin E2 activates mitogen-activated protein kinase/Erk pathway signaling and cell proliferation in non-small cell lung cancer cells in an epidermal growth factor receptor-independent manner. Cancer Res.

[15] Dohadwala M (2006). Cyclooxygenase-2-dependent regulation of E-cadherin: prostaglandin E(2) induces transcriptional repressors ZEB1 and snail in non-small cell lung cancer. Cancer Res.

[16] Pold M (2004). Cyclooxygenase-2-dependent expression of angiogenic CXC chemokines ENA-78/CXC Ligand (CXCL) 5 and interleukin-8/CXCL8 in human non-small cell lung cancer. Cancer Res.

[17] St John MA (2009). Proinflammatory mediators upregulate snail in head and neck squamous cell carcinoma. Clin Cancer Res.

[18] Lee JM (2008). Inflammation in lung carcinogenesis: new targets for lung cancer chemoprevention and treatment. Crit Rev Oncol Hematol.

[19] Masola V (2019). *In vitro* effects of interleukin (IL)-1 beta inhibition on the epithelial-to-mesenchymal transition (EMT) of renal tubular and hepatic stellate cells. J Transl Med.

[20] Wu T (2016). Modulation of IL-1beta reprogrammes the tumor microenvironment to interrupt oral carcinogenesis. Sci Rep.

[21] Fidler IJ (2003). The pathogenesis of cancer metastasis: the ‘seed and soil’ hypothesis revisited. Nat Rev Cancer.

[22] Chambers AF, Groom AC, MacDonald IC (2002). Dissemination and growth of cancer cells in metastatic sites. Nat Rev Cancer.

[23] Thiery JP (2002). Epithelial-mesenchymal transitions in tumour progression. Nat Rev Cancer.

[24] Ye X, Weinberg RA (2015). Epithelial-Mesenchymal Plasticity: A Central Regulator of Cancer Progression. Trends Cell Biol.

[25] Giannou AD (2015). Mast cells mediate malignant pleural effusion formation. J Clin Invest.

[26] Walser TC (2018). Silencing the Snail-Dependent RNA Splice Regulator ESRP1 Drives Malignant Transformation of Human Pulmonary Epithelial Cells. Cancer Res.

[27] Tsai JH, Donaher JL, Murphy DA, Chau S, Yang J (2012). Spatiotemporal regulation of epithelial-mesenchymal transition is essential for squamous cell carcinoma metastasis. Cancer Cell.

[28] Tam WL, Weinberg RA (2013). The epigenetics of epithelial-mesenchymal plasticity in cancer. Nat Med.

[29] Lou Y (2016). Epithelial-Mesenchymal Transition Is Associated with a Distinct Tumor Microenvironment Including Elevation of Inflammatory Signals and Multiple Immune Checkpoints in Lung Adenocarcinoma. Clin Cancer Res.

[30] Chae YK (2018). Epithelial-mesenchymal transition (EMT) signature is inversely associated with T-cell infiltration in non-small cell lung cancer (NSCLC). Sci Rep.

[31] Liberzon A (2015). The Molecular Signatures Database (MSigDB) hallmark gene set collection. Cell Syst.

[32] Javaid S (2013). Dynamic chromatin modification sustains epithelial-mesenchymal transition following inducible expression of Snail-1. Cell Rep.

[33] Dong C (2013). Interaction with Suv39H1 is critical for Snail-mediated E-cadherin repression in breast cancer. Oncogene.

[34] Dong C (2012). G9a interacts with Snail and is critical for Snail-mediated E-cadherin repression in human breast cancer. J Clin Invest.

[35] Schwartsmann G (2000). A phase I trial of cisplatin plus decitabine, a new DNA-hypomethylating agent, in patients with advanced solid tumors and a follow-up early phase II evaluation in patients with inoperable non-small cell lung cancer. Invest New Drugs.

[36] Greten FR, Grivennikov SI (2019). Inflammation and Cancer: Triggers, Mechanisms, and Consequences. Immunity.

[37] Katsuno Yoko, Meyer Dominique Stephan, Zhang Ziyang, Shokat Kevan M., Akhurst Rosemary J., Miyazono Kohei, Derynck Rik (2019). Chronic TGF-β exposure drives stabilized EMT, tumor stemness, and cancer drug resistance with vulnerability to bitopic mTOR inhibition. Science Signaling.

[38] Marazioti A (2018). Myeloid-derived interleukin-1beta drives oncogenic KRAS-NF-kappaBeta addiction in malignant pleural effusion. Nat Commun.

[39] Szczerba BM (2019). Neutrophils escort circulating tumour cells to enable cell cycle progression. Nature.

[40] Du J, Johnson LM, Jacobsen SE, Patel DJ (2015). DNA methylation pathways and their crosstalk with histone methylation. Nat Rev Mol Cell Biol.

[41] Cedar H, Bergman Y (2009). Linking DNA methylation and histone modification: patterns and paradigms. Nat Rev Genet.

[42] Meissner A (2008). Genome-scale DNA methylation maps of pluripotent and differentiated cells. Nature.

[43] Epsztejn-Litman S (2008). De novo DNA methylation promoted by G9a prevents reprogramming of embryonically silenced genes. Nat Struct Mol Biol.

[44] Schlesinger Y (2007). Polycomb-mediated methylation on Lys27 of histone H3 pre-marks genes for de novo methylation in cancer. Nat Genet.

[45] Mohn F (2008). Lineage-specific polycomb targets and de novo DNA methylation define restriction and potential of neuronal progenitors. Mol Cell.

[46] Vire E (2006). The Polycomb group protein EZH2 directly controls DNA methylation. Nature.

[47] Rougeulle C (2004). Differential histone H3 Lys-9 and Lys-27 methylation profiles on the X chromosome. Mol Cell Biol.

[48] Mozzetta C (2014). The histone H3 lysine 9 methyltransferases G9a and GLP regulate polycomb repressive complex 2-mediated gene silencing. Mol Cell.

[49] Bintu L (2016). Dynamics of epigenetic regulation at the single-cell level. Science.

[50] Juergens RA (2011). Combination epigenetic therapy has efficacy in patients with refractory advanced non-small cell lung cancer. Cancer Discov.

[51] Topalian SL (2012). Safety, activity, and immune correlates of anti-PD-1 antibody in cancer. N Engl J Med.

[52] Brahmer J (2015). Nivolumab versus Docetaxel in Advanced Squamous-Cell Non-Small-Cell Lung Cancer. N Engl J Med.

[53] Topper MJ (2017). Epigenetic Therapy Ties MYC Depletion to Reversing Immune Evasion and Treating Lung Cancer. Cell.

[54] Xu Shili, Catapang Arthur, Doh Hanna M., Bayley Nicholas A., Lee Jason T., Braas Daniel, Graeber Thomas G., Herschman Harvey R. (2018). Hexokinase 2 Is Targetable for HK1-Negative, HK2-Positive Tumors from a Wide Variety of Tissues of Origin. Journal of Nuclear Medicine.

[55] Pagano PC (2017). Identification of a Human Airway Epithelial Cell Subpopulation with Altered Biophysical, Molecular, and Metastatic Properties. Cancer Prev Res (Phila).

[56] Liclican EL (2014). Loss of miR125a expression in a model of K-ras-dependent pulmonary premalignancy. Cancer Prev Res (Phila).

[57] Graff JR, Herman JG, Myohanen S, Baylin SB, Vertino PM (1997). Mapping patterns of CpG island methylation in normal and neoplastic cells implicates both upstream and downstream regions in de novo methylation. J Biol Chem.

[58] Anders S, Pyl PT, Huber W (2015). HTSeq–a Python framework to work with high-throughput sequencing data. Bioinformatics.

[59] Hanzelmann S, Castelo R, Guinney J (2013). GSVA: gene set variation analysis for microarray and RNA-seq data. BMC Bioinformatics.

[60] Conesa A, Nueda MJ, Ferrer A, Talon M (2006). maSigPro: a method to identify significantly differential expression profiles in time-course microarray experiments. Bioinformatics.

