# Author Correction: Chronic IL-1β-induced inflammation regulates epithelial-to-mesenchymal transition memory phenotypes via epigenetic modifications in non-small cell lung cancer

**DOI:** 10.1038/s41598-020-61341-3

**Published:** 2020-03-04

**Authors:** Rui Li, Stephanie L. Ong, Linh M. Tran, Zhe Jing, Bin Liu, Stacy J. Park, Zi Ling Huang, Tonya C. Walser, Eileen L. Heinrich, Gina Lee, Ramin Salehi-Rad, William P. Crosson, Paul C. Pagano, Manash K. Paul, Shili Xu, Harvey Herschman, Kostyantyn Krysan, Steven Dubinett

**Affiliations:** 1 Pulmonary and Critical Care Medicine, David Geffen School of Medicine at UCLA, Los Angeles, 90025 California USA; 2 Department of Molecular and Medical Pharmacology, David Geffen School of Medicine at UCLA, Los Angeles, 90025 California USA; 3 Department of Medicine, David Geffen School of Medicine at UCLA, Los Angeles, 90025 California USA; 4 Department of Pathology and Laboratory Medicine, David Geffen School of Medicine at UCLA, Los Angeles, 90025 California USA; 5 VA Greater Los Angeles Health Care System, Los Angeles, California 90025 USA

Correction to: *Scientific Reports* 10.1038/s41598-019-57285-y, published online 15 January 2020

In Figure 6E, the label ‘Decitabine’ was inadvertently included. The correct Figure 6 appears below as Figure 1.

**Figure 1:**
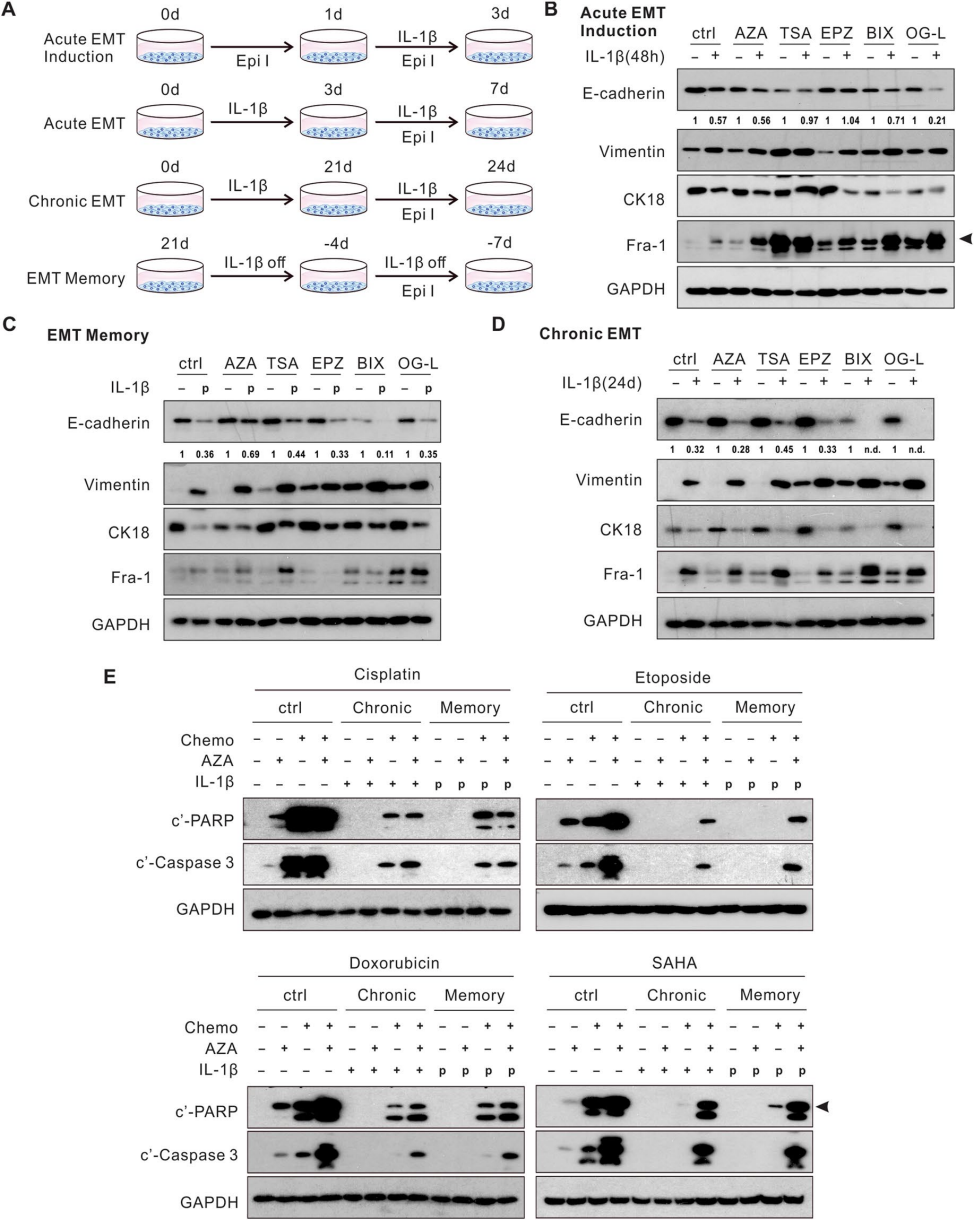
.

