# Supplementary material for: Chronic IL-1β-induced inflammation regulates epithelial-to-mesenchymal transition memory phenotypes via epigenetic modifications in non-small cell lung cancer
Source: Sci Rep. 2020 Jan 15;10:377. doi: 10.1038/s41598-019-57285-y (PMC6962381; doi:10.1038/s41598-019-57285-y)
Supplement: Supplementary file 1 — Dataset 1. [file 41598_2019_57285_MOESM1_ESM.pdf]

Chronic IL-1β-induced inflammation regulates epithelial-to-mesenchymal transition memory phenotypes via epigenetic modification in non-small cell lung cancer

Rui Li[1,2], Stephanie L. Ong[3], Linh M. Tran[1], Zhe Jing[1], Bin Liu[1], Stacy J. Park[1], Zi Ling Huang[1], Tonya C. Walser[1], Eileen L. Heinrich[4], Gina Lee[1,5], Ramin Salehi-Rad[1,5], William P. Crosson[2], Paul C. Pagano[2], Manash K. Paul[1], Shili Xu[2], Harvey Herschman[2], Kostyantyn Krysan[1], Steven Dubinett[1,2,3,4,5,*]

[1]Pulmonary and Critical Care Medicine, David Geffen School of Medicine at UCLA, Los Angeles, 90025, California, USA

[2]Department of Molecular and Medical Pharmacology, David Geffen School of Medicine at UCLA, Los Angeles, 90025, California, USA

[3]Department of Medicine, David Geffen School of Medicine at UCLA, Los Angeles, 90025, California, USA

[4]Department of Pathology and Laboratory Medicine, David Geffen School of Medicine at UCLA, Los Angeles, 90025, California, USA

[5]VA Greater Los Angeles Health Care System, Los Angeles, California, 90025, USA

*Correspondence: sdubinett@mednet.ucla.edu

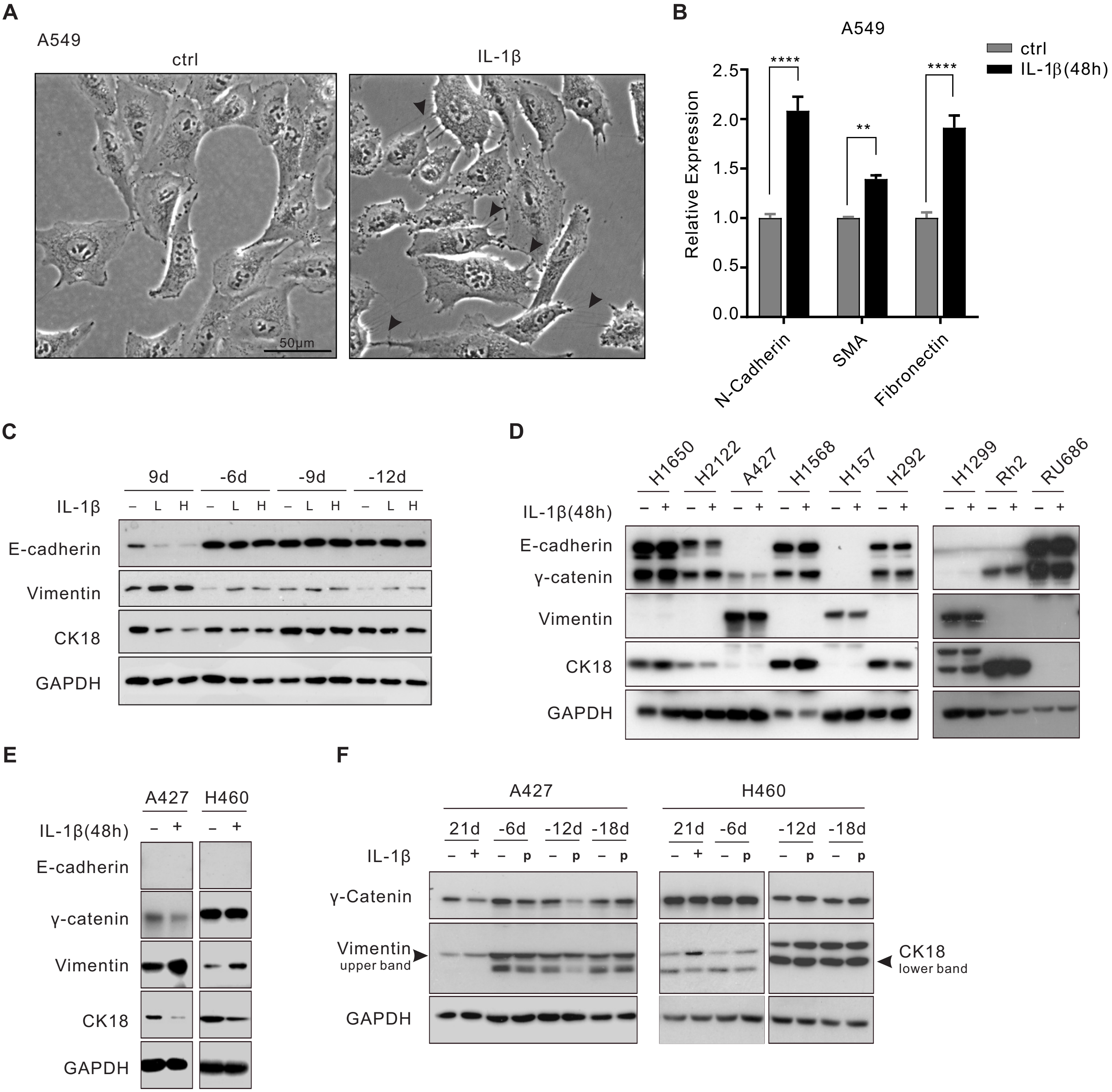
A
A549
ctrl
IL-1β
50μm
B
A549
ctrl
IL-1β(48h)
Relative Expression
2.5
2.0
1.5
1.0
0.5
0.0
****
**
****
N-Cadherin
SMA
Fibronectin
C
9d
-6d
-9d
-12d
IL-1β
– L H – L H – L H – L H
E-cadherin
Vimentin
CK18
GAPDH
D
H1650
H2122
A427
H1568
H157
H292
H1299
Rh2
RU686
IL-1β(48h)
– + – + – + – + – + – + – + – + – +
E-cadherin
γ-catenin
Vimentin
CK18
GAPDH
E
A427
H460
IL-1β(48h)
– + – +
E-cadherin
γ-catenin
Vimentin
CK18
GAPDH
F
A427
H460
21d
-6d
-12d
-18d
IL-1β
– + – p – p – p
– + – p – p – p
γ-Catenin
Vimentin
upper band
CK18
lower band
GAPDH

Figure S1

Figure S1 related to Figure 1. Chronic cytokine exposure leads to EMT memory in NSCLC. **A**, Phase-contrast microscopy showed decreased cell-to-cell contacts and increased cell protrusions (indicated by arrow head) following 48-hour IL-1β exposure in A549 cells. **B**, Gene expression of indicated mesenchymal markers by RT-PCR. **C**, High dose of IL-1β exposure did not lead to EMT memory following 9-day IL-1β exposure in A549 cells. **D**, A screening of EMT in indicated NSCLC cell lines following 48-hour IL-1β treatment. **E**, EMT markers in A427 and H460 cell lines following 48-hour IL-1β exposure. E-cadherin was not detected in these two cell lines. **F**, Prolonged EMT phenotype was observed in A427 and H460 following chronic IL-1β exposure. L, low dose (1 ng/ml); H, high dose (10 ng/ml). "p", previously treated with IL-1β. All results were reported as mean ± SEM. ** P<0.002, **** P<0.0001.

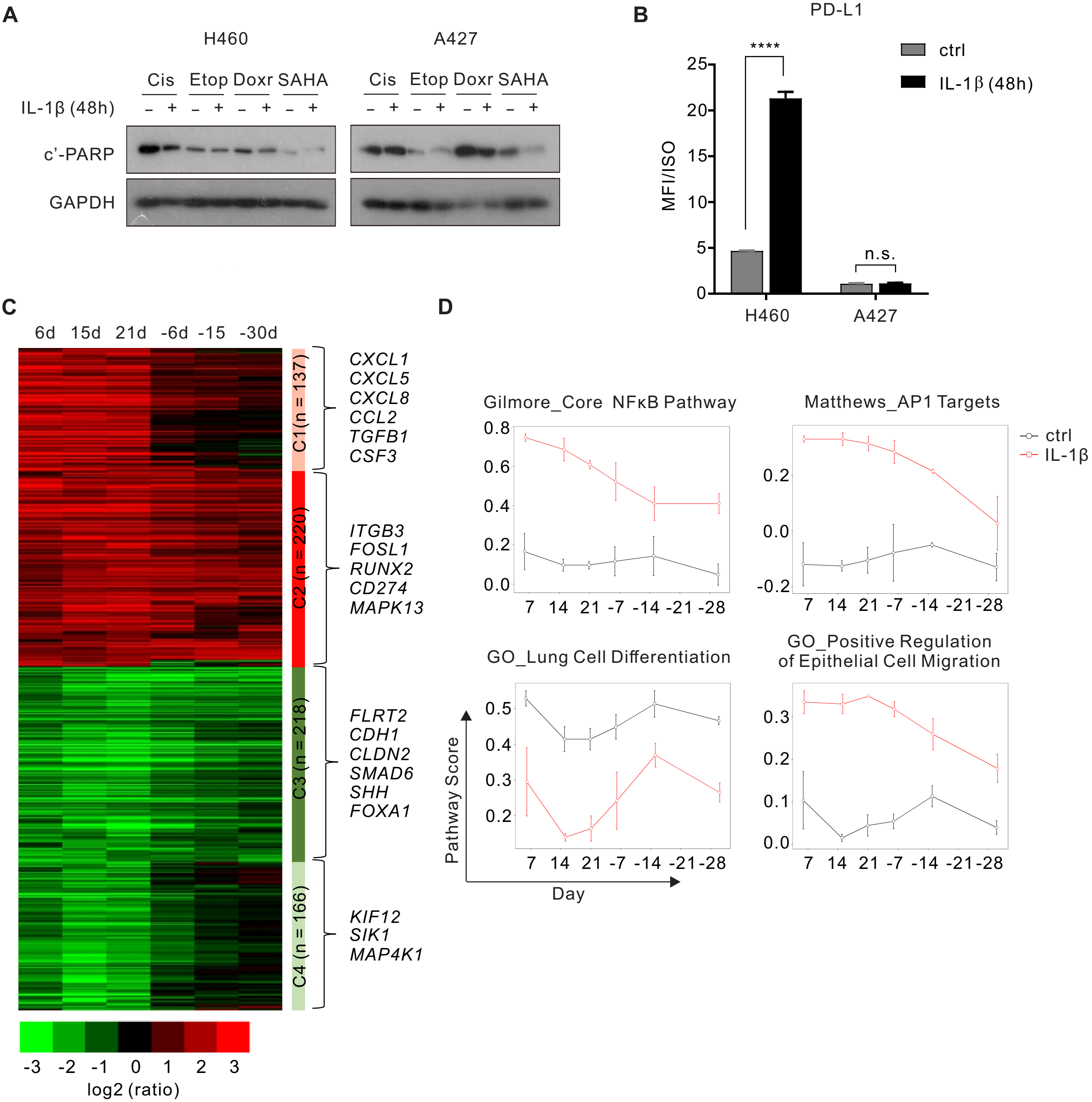
A
H460
A427
Cis
Etop
Doxr
SAHA
IL-1β (48h)
c'-PARP
GAPDH
B
PD-L1
ctrl
IL-1β (48h)
MFI/ISO
****
n.s.
H460
A427
C
6d 15d 21d -6d -15 -30d
C1 (n = 137)
C2 (n = 220)
C3 (n = 218)
C4 (n = 166)
CXCL1
CXCL5
CXCL8
CCL2
TGFB1
CSF3
ITGB3
FOSL1
RUNX2
CD274
MAPK13
FLRT2
CDH1
CLDN2
SMAD6
SHH
FOXA1
KIF12
SIK1
MAP4K1
log2 (ratio)
D
Gilmore_Core NFκB Pathway
Matthews_AP1 Targets
GO_Lung Cell Differentiation
GO_Positive Regulation of Epithelial Cell Migration
ctrl
IL-1β
Pathway Score
Day

Figure S2

Figure S2 related to Figure 2. EMT-associated phenotypes induced by chronic IL-1β exposure are also memorized. **A**, Chemotherapy-induced apoptosis following the acute IL-1β exposure in H460 and A427 cells. **B**, Surface PD-L1 expression following the acute IL-1β exposure in H460 and A427 cells. **C**, A heatmap of differentially expressed genes at indicated time points following IL-1β exposure, which were divided into four clusters. C1 and C4, genes sensitive to IL-1β exposure; C2 and C3, genes memorizing their expression patterns following IL-1β withdrawal. **D**, Pathway analysis in A549 cells showing that pathways, such as the NF-κB and AP-1 pathways, and phenotypes, such as cell differentiation and migration, are IL-1β sensitive**.** Cis, cisplatin; Etop, etoposide; Doxr, doxorubicin. All results were reported as mean ± SEM. **** $P < 0.0001$, n.s, not significant.

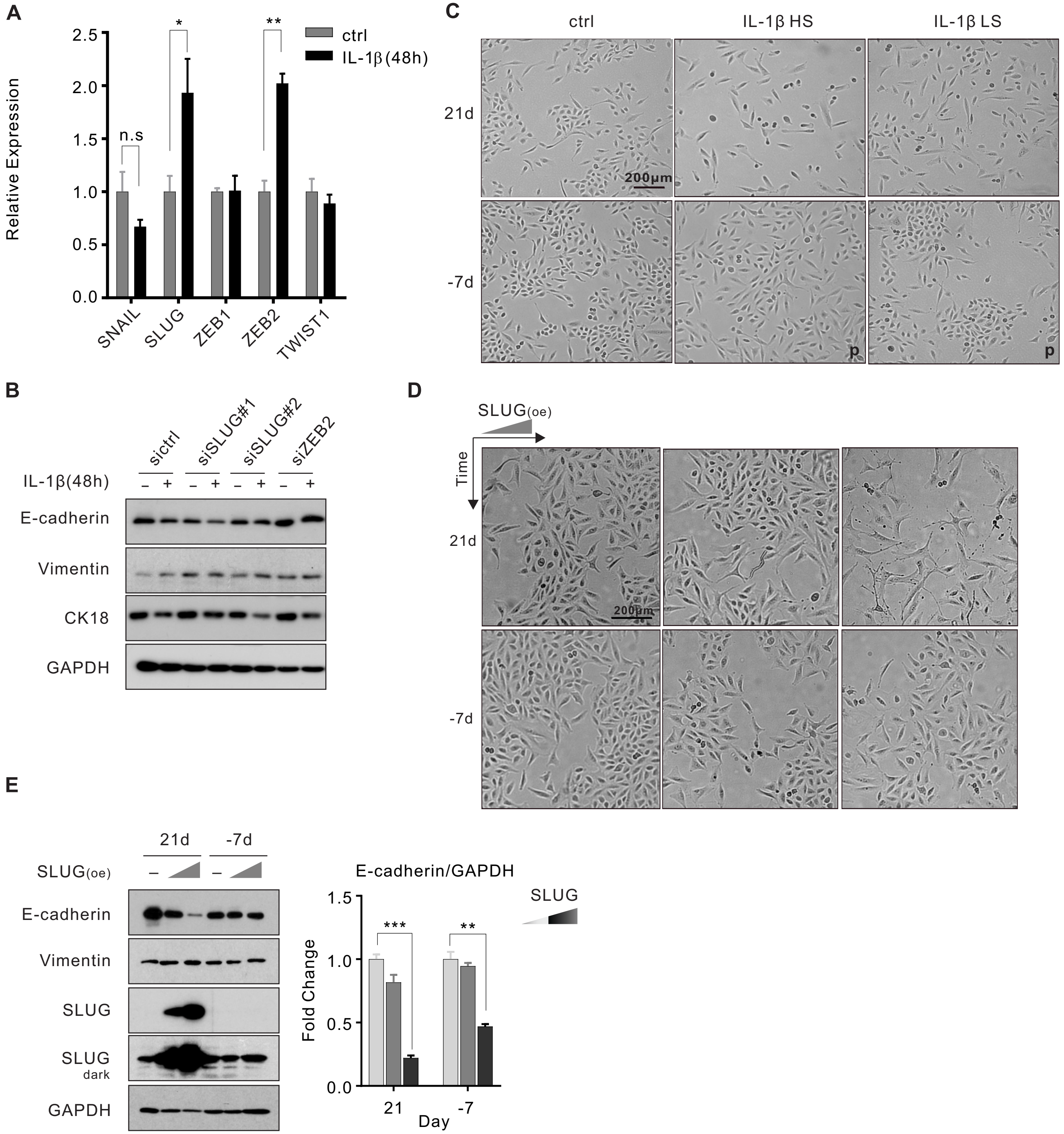
A
Relative Expression
ctrl
IL-1β (48h)
n.s
*
**
SNAIL
SLUG
ZEB1
ZEB2
TWIST1
B
sictrl
siSLUG#1
siSLUG#2
siZEB2
IL-1β(48h)
E-cadherin
Vimentin
CK18
GAPDH
C
ctrl
IL-1β HS
IL-1β LS
21d
-7d
200μm
D
SLUG(oe)
Time
21d
-7d
200μm
E
21d
-7d
SLUG(oe)
E-cadherin
Vimentin
SLUG
SLUG dark
GAPDH
E-cadherin/GAPDH
SLUG
Fold Change
***
**
Day

Figure S3

F

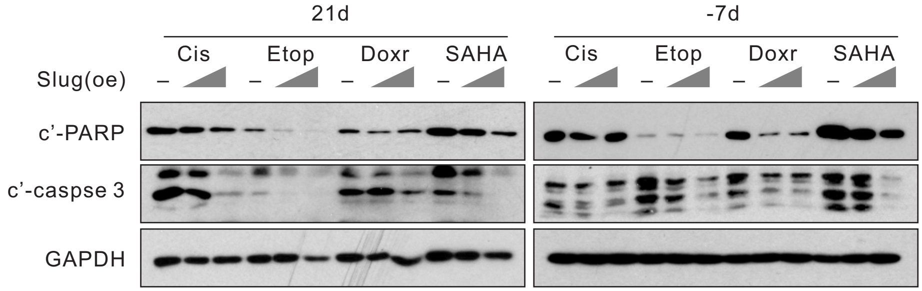
21d
-7d
Cis
Etop
Doxr
SAHA
Slug(oe)
c'-PARP
c'-caspse 3
GAPDH

G

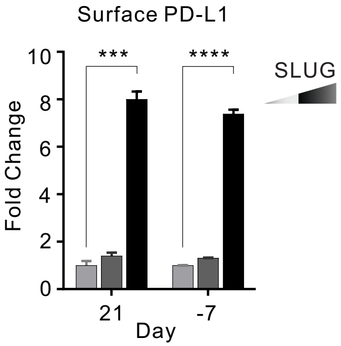
Surface PD-L1
Fold Change
10
8
6
4
2
0
***
****
SLUG
21
-7
Day

Figure S3

Figure S3 related to Figure 3. Accumulation of SLUG is indispensable for the establishment of the memory phenotypes. A, Relative expression of EMT-associated transcription repressors by RT-qPCR following the acute IL-1β exposure. **B**, Knockdown of SLUG or ZEB2 is sufficient to prevent E-cadherin downregulation following the acute IL-1β exposure. **C**, Morphological changes of the untreated, LS, and HS cells by bright-field microscopy. **D**, Morphological changes with various levels of SLUG at day 21 of SLUG overexpression (21d) and day 7 after the overexpression was terminated (-7d). **E-G**, Evaluation of EMT markers, apoptosis resistance, and surface PD-L1 expression with various levels of SLUG overexpression at indicated time points. "p", previously treated with IL-1β. "oe", overexpression. Cis, cisplatin; Etop, etoposide; Doxr, doxorubicin. All results were reported as mean ± SEM. * $P<0.03$, ** $P<0.002$, *** $P<0.0002$, **** $P<0.0001$, n.s, not significant.

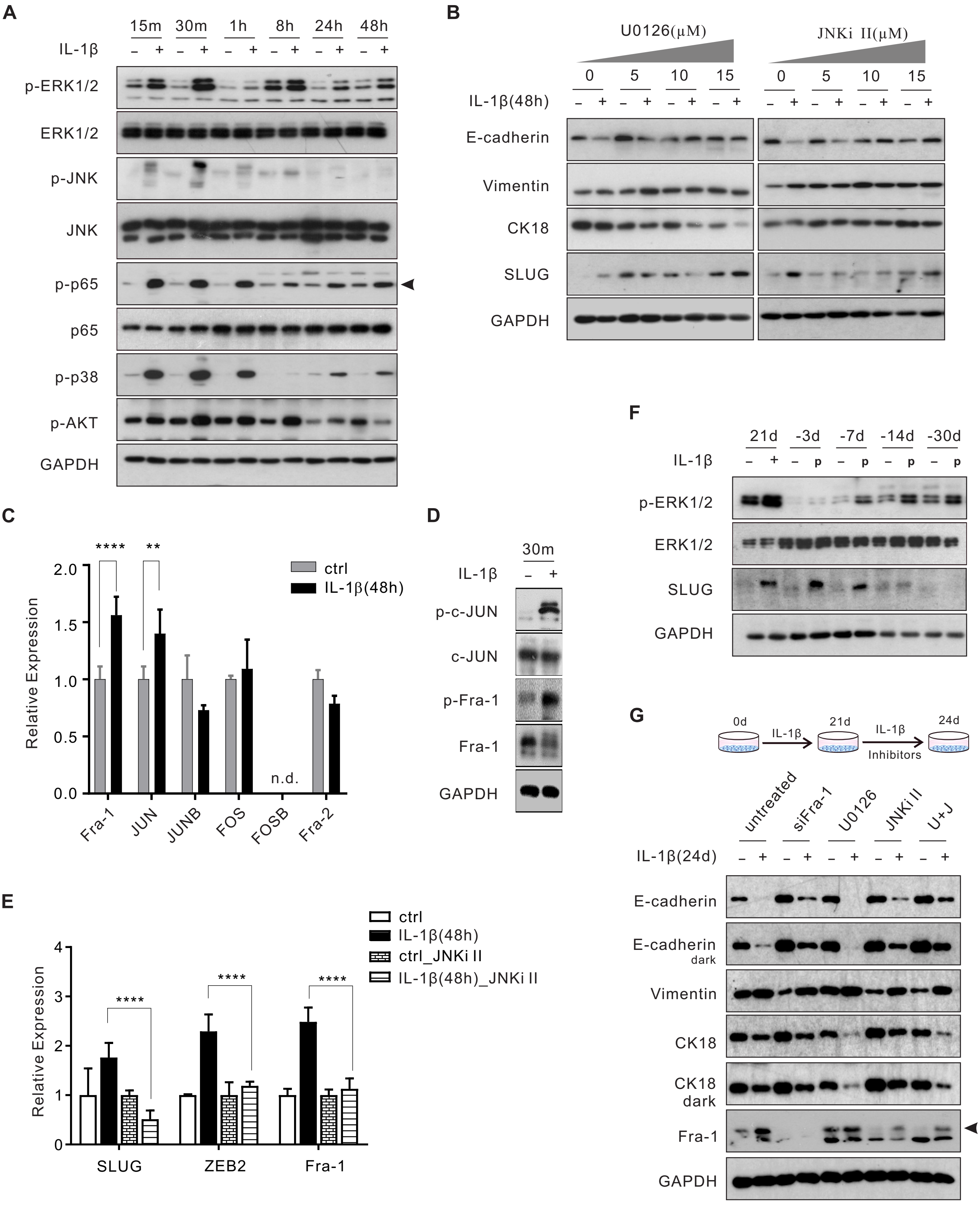

A
15m
30m
1h
8h
24h
48h
IL-1β
p-ERK1/2
ERK1/2
p-JNK
JNK
p-p65
p65
p-p38
p-AKT
GAPDH
B
U0126(μM)
JNKi II(μM)
0
5
10
15
IL-1β(48h)
E-cadherin
Vimentin
CK18
SLUG
GAPDH
C
ctrl
IL-1β(48h)
Relative Expression
n.d.
Fra-1
JUN
JUNB
FOS
FOSB
Fra-2
D
30m
IL-1β
p-c-JUN
c-JUN
p-Fra-1
Fra-1
GAPDH
E
ctrl
IL-1β(48h)
ctrl_JNKi II
IL-1β(48h)_JNKi II
Relative Expression
SLUG
ZEB2
Fra-1
F
21d
-3d
-7d
-14d
-30d
IL-1β
p-ERK1/2
ERK1/2
SLUG
GAPDH
G
0d
IL-1β
21d
IL-1β
Inhibitors
24d
untreated
siFra-1
U0126
JNKi II
U+J
IL-1β(24d)
E-cadherin
E-cadherin dark
Vimentin
CK18
CK18 dark
Fra-1
GAPDH

Figure S4

Figure S4 related to Figure 4. Pathways mediating acute EMT are not required for the maintenance of chronic EMT or EMT memory. A, Signaling pathways activated by IL-1β within 48 hours in A549 cells. **B**, A dose-dependent response of E-cadherin expression to the ERK or JNK inhibitor in acute EMT. **C**, Expression levels of the transcription factor AP-1 components by RT-qPCR at 48 hours following the IL-1β treatment. **D**, Phosphorylation of Fra-1 and c-JUN upon 30 minutes of IL-1β exposure, examined by immunoblotting. **E**, Relative expression of Fra-1, SLUG and ZEB2 with the JNK inhibition followed by the acute IL-1β exposure by RT-qPCR. **F**, Persistent activation of the ERK pathway in chronic EMT and EMT memory. **G**, EMT markers following 72-hour inhibition of the MAPK (ERK and JNK) - AP - 1 - SLUG axis in chronic EMT, examined by immunoblotting. U0126, ERK pathway inhibitor; JNKi II, JNK inhibitor; "U+J", U0126 and JNKi II combination treatment. "-d", days after IL-1β withdrawal; "p", previously treated with IL-1β. "n.d.", non-detectable. All results were reported as mean ± SEM. ** $P<0.002$, **** $P<0.0001$, N.D., not detectable.

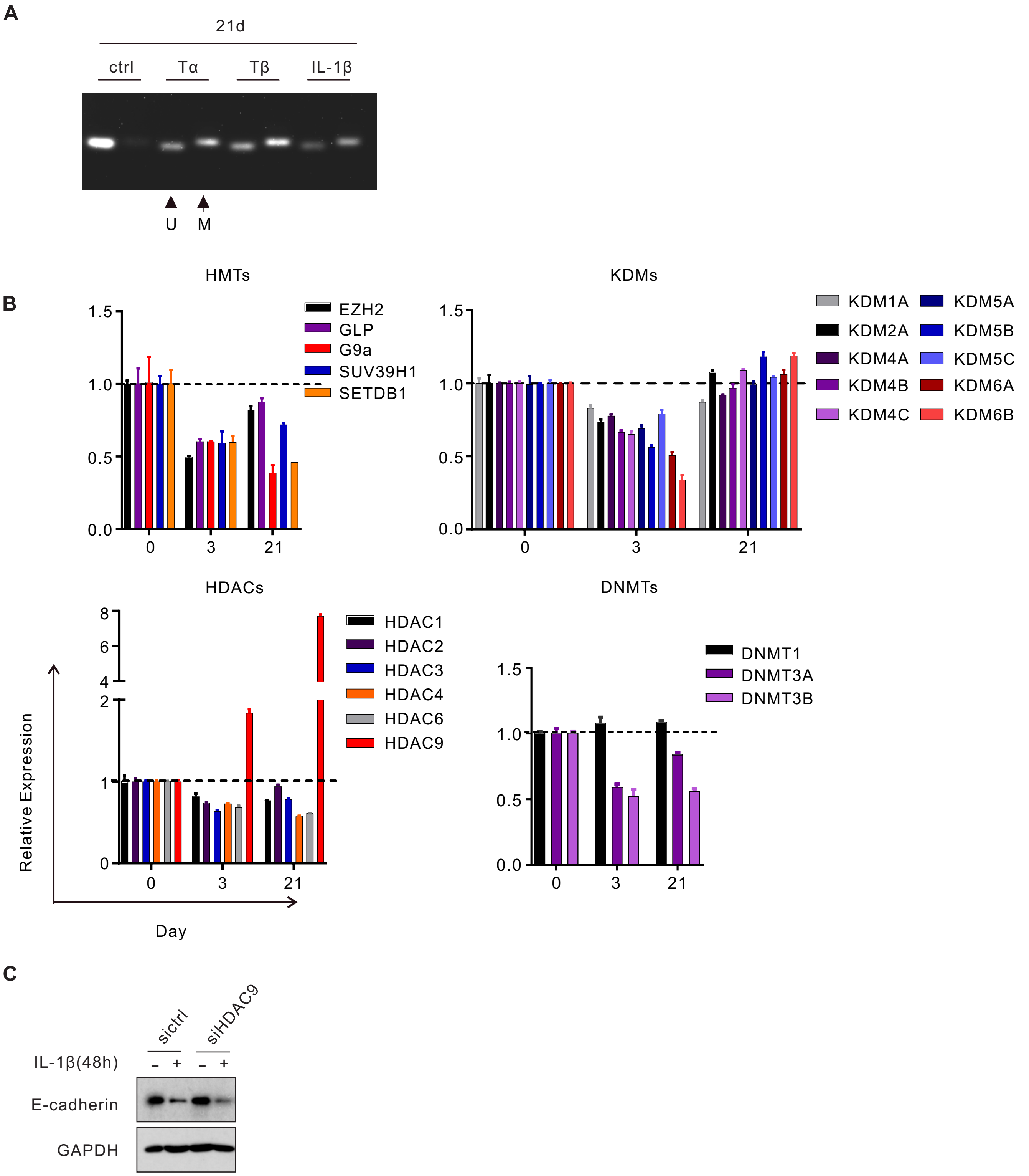
A
21d
ctrl
Tα
Tβ
IL-1β
U
M
B
HMTs
1.5
1.0
0.5
0.0
0
3
21
EZH2
GLP
G9a
SUV39H1
SETDB1
KDMs
KDM1A
KDM2A
KDM4A
KDM4B
KDM4C
KDM5A
KDM5B
KDM5C
KDM6A
KDM6B
HDACs
8
6
4
2
1
0
HDAC1
HDAC2
HDAC3
HDAC4
HDAC6
HDAC9
DNMTs
DNMT1
DNMT3A
DNMT3B
Relative Expression
Day
C
sictrl
siHDAC9
IL-1β(48h)
–
+
E-cadherin
GAPDH

Figure S5

Figure S5 related to Figure 5. Dynamic epigenetic modifications of the *CDH1* promoter in IL-1β-induced EMT. **A**, Increased DNA methylation in the *CDH1* promoter upon the chronic TNF-α (10 ng/ml) or TGF-β (5 ng/ml) exposure. IL-1β served as a positive control. **B**, RT-qPCR revealed the expression levels of various histone methyltransferases (HMTs), lysine demethylases (KDMs), histone deacetylases (HDACs), and DNA methyltransferases (DNMTs) following the IL-1β treatment at indicated time points. **C**, HDAC9 knockdown did not affect E-cadherin expression in acute EMT. Tα, TNF-α; Tβ, TGF-β. M, methylated; U, unmethylated.

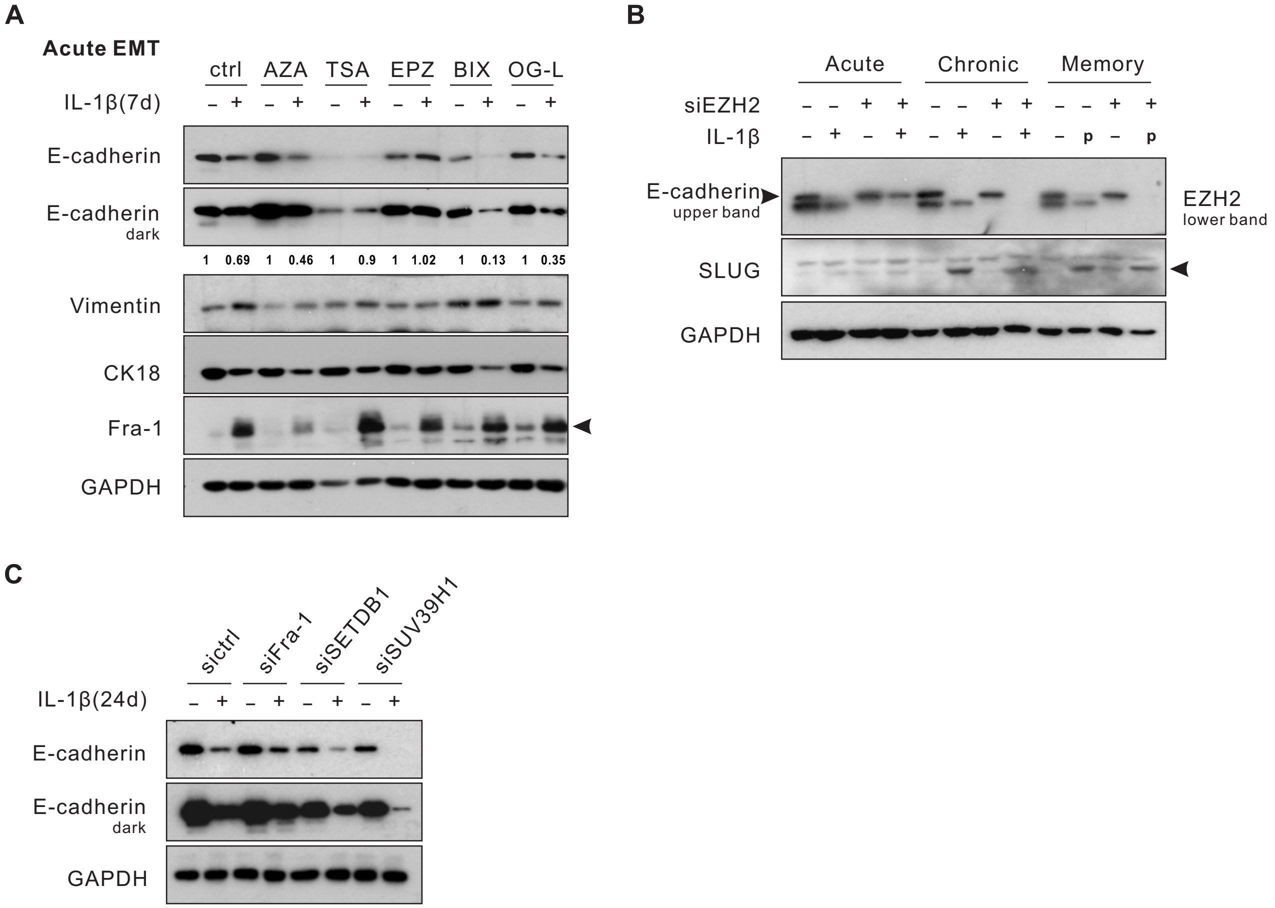
A
Acute EMT
ctrl
AZA
TSA
EPZ
BIX
OG-L
IL-1β(7d)
E-cadherin
E-cadherin
dark
1 0.69 1 0.46 1 0.9 1 1.02 1 0.13 1 0.35
Vimentin
CK18
Fra-1
GAPDH
B
Acute
Chronic
Memory
siEZH2
IL-1β
E-cadherin
upper band
EZH2
lower band
SLUG
GAPDH
C
sictrl
siFra-1
siSETDB1
siSUV39H1
IL-1β(24d)
E-cadherin
E-cadherin
dark
GAPDH

Figure S6

Figure S6 related to Figure 6. Blockade of epigenetic modifications restores E-cadherin expression and cellular sensitivity to chemotherapy in EMT memory. A, Epigenetic inhibitors were added following 4 days of IL-1β exposure. TSA and EPZ reversed the downregulation of E-cadherin in acute EMT. Quantification of E-cadherin expression was normalized to samples without IL-1β exposure. **B**, E-cadherin expression was evaluated following EZH2 knockdown for 72 hours in acute, chronic EMT, and EMT memory. **C**. E-cadherin expression in chronic EMT following SETDB1 or SUV39H1 knockdown for 72 hours. AZA, 5'-azacytidine-2′-deoxycytidine (decitabine), DNA methyltransferase inhibitor; TSA, pan HDAC inhibitor; EPZ, EPZ-6438, EZH2 inhibitor; BIX, BIX01294, G9a inhibitor; OG-L, OG-L002, LSD1 inhibitor. "p", previously treated with IL-1β.

Table S1 related to Figure 2. EMT-associated phenotypes induced by chronic IL-1β exposure are also memorized. Differentially expressed gene upon IL-1β exposure.

Table S2 related to Figure 2. EMT-associated phenotypes induced by chronic IL-1β exposure are also memorized. Pathway analysis based on the Molecular Signature Database.
